# Large language models outperform mental and medical health care professionals in identifying obsessive-compulsive disorder

**DOI:** 10.1038/s41746-024-01181-x

**Published:** 2024-07-19

**Authors:** Jiyeong Kim, Kimberly G. Leonte, Michael L. Chen, John B. Torous, Eleni Linos, Anthony Pinto, Carolyn I. Rodriguez

**Affiliations:** 1 Stanford Center for Digital Health, Department of Medicine, Stanford University, Palo Alto, CA USA; 2 Clearview Horizons, North Andover, MA USA; 3 Division of Digital Psychiatry, Department of Psychiatry, Beth Israel Deaconess Medical Center, Boston, MA USA; 4 Department of Psychiatry, Donald and Barbara Zucker School of Medicine at Hofstra/Northwell, Hempstead, NY USA; 5 Northwell, New Hyde Park, NY USA; 6 Department of Psychiatry and Behavioral Sciences, School of Medicine, Stanford University, Palo Alto, CA USA; 7 Veterans Affairs Palo Alto Health Care System, Palo Alto, CA USA

**Keywords:** Translational research, Psychiatric disorders, Diagnosis

## Abstract

Despite the promising capacity of large language model (LLM)-powered chatbots to diagnose diseases, they have not been tested for obsessive-compulsive disorder (OCD). We assessed the diagnostic accuracy of LLMs in OCD using vignettes and found that LLMs outperformed medical and mental health professionals. This highlights the potential benefit of LLMs in assisting in the timely and accurate diagnosis of OCD, which usually entails a long delay in diagnosis and treatment.

Large language model (LLM)-powered artificial intelligence (AI) chatbots exhibit professional-level knowledge across multiple medical specialty areas^1,2^ and have been evaluated for disease detection, treatment suggestions, medical education, and triage assistance^3,4^. Moreover, their ability in advanced clinical reasoning holds promise in assisting in a physician’s diagnosis and treatment planning^5–7^. In a statement from the American Psychiatric Association (APA), caution was urged in the use of LLM tools in clinical decision-making^8^; yet a recent survey revealed that many psychiatrists use LLMs in answering clinical questions and documenting notes, and they believe that LLMs would improve diagnostic accuracy in psychiatry^9^. Given the interest and current usage, rigorous study of these tools is urgently needed, especially with respect to awareness of potential bias, compliance with HIPAA, and the use of LLM to augment decision-making.

Obsessive-compulsive disorder (OCD) is a common mental health condition, doing repetitive behaviors (compulsions) to avoid unwanted thoughts or sensations (obsessions), which significantly disrupt the daily lives of a person and the family^10^. It affects approximately 1 in 40 adults in the United States^11^, and among adults with OCD, nearly one-half experience serious disability^12^. Unfortunately, there is, on average, a 17-year delay between the onset of symptoms and treatment initiation^13^. Longer duration of untreated illness has a negative impact on the long-term outcome of patients with OCD in terms of inferior treatment response and increased symptom severity^14^. Although there have been efforts to detect mental disorders through social media and online languages, exploration of LLMs in OCD identification has been limited despite their potential contribution to improving diagnostic delay^9,15–17^. Given the patient data safety concerns around deploying LLMs in care, recent studies have employed clinical vignettes for LLM assessments^18,19^. The differing outcomes around the performance of LLMs for mental health studies^17^ highlight the need for rigorous study design that can encompass the performance variability of LLMs, including testing multiple LLMs. Given that to our knowledge this method has not been tested in OCD, it is critical to explore the potential of LLM to diagnose OCD. Thus, the goal of this study was to examine the diagnostic accuracy of LLM compared to clinicians and other mental health professionals using clinical vignettes of OCD.

One LLM (ChatGPT-4) correctly ranked OCD as the primary diagnosis in all responded case vignettes (100%, *N* = 16/16 [*n* = 4/4 in 2013 vignettes; *n* = 7/7 in 2015 vignettes; *n* = 5/5 in 2022 vignettes]) (see Table 1). ChatGPT-4 and Gemini Pro both did not provide a response for OCD vignettes with sexual content, noting “content violation”^20^. Notably, the overall LLM diagnostic accuracy (96.1%, *N* = 49/51) was higher than that of medical and mental health professionals in prior studies. The most accurate group of clinicians was doctoral trainees in psychology (81.5%, *N* = 106/130). Moreover, the overall accuracy of LLMs for OCD identification was more than 30% higher than that of primary care physicians (49.5%) and more than 20% higher than that of American Psychological Association members (61.1%). Two other LLMs missed one diagnosis each while they showed higher accuracy than all other mental health providers in this study (Llama 3: 94.7%, *n* = 18/19; Gemini Pro: 93.8%, *n* = 15/16). Llama 3 could not differentiate sexual orientation obsessions and sexual identity confusion, and Gemini Pro could not differentiate somatic obsessions and body dysmorphic disorder (see Table S3). For control vignettes, the overall diagnostic accuracy of three LLMs was 100.0% (*N* = 21/21), presenting consistency across the LLMs (ChatGPT-4: *n* = 7/7; Gemini Pro: *n* = 7/7; Llama 3: *n* = 7/7) (see Fig. 1). One incorrect reasoning was identified in Llama 3 and Gemini Pro each while ChatGPT-4’s reasoning was all correct. Cohen’s kappa was 1.0 (perfect agreement) (see Supplementary Table 2).

**Table 1:** OCD identification rate of LLMs and medical and mental health professionals

Content of OCD vignette	LLM			Group 1. APA members^a^ (*N* = 360)	Group 2. Primary care physicians^b^ (*N* = 208)	Group 3. Doctoral trainees in psychology^c^ (*N* = 130)	Group 4. Medical providers in Guam^d^ (*N* = 105)	Group 5. Clergy members in Guam (*N* = 110)
	ChatGPT-4 (*N* = 16)	Gemini Pro (*N* = 16)	Llama 3 (*N* = 19)					
Across all vignettes	96.1% (*N* = 49/51)^e^			61.1%	49.5%	81.5%	41.9%	35.5%
	100% (*n* = 16/16)	93.8% (*n* = 15/16)	94.7% (*n* = 18/19)					
Vignette 1. Harm obsessions	100% (3/3 trials)	100% (3/3 trials)	100% (3/3 trials)	68.5%	20.0%	77.8%	18.2%	27.8%
Vignette 2. Sexual orientation obsessions	100% (3/3 trials)	100% (2/2 trials)^g^	66.7% (2/3 trials)	23.0%	15.4%	66.7%	10.0%	6.7%
Vignette 3. Sexual attraction to children obsessions	No response^f^	100% (1/1 trial)^h^	100% (3/3 trails)	57.1%	29.2%	77.8%	15.0%	11.1%
Vignette 4. Religious obsessions	100% (3/3 trials)	100% (3/3 trials)	100% (3/3 trials)	71.2%	62.5%	80.0%	72.7%	21.7%
Vignette 5. Contamination obsessions	100% (3/3 trials)	100% (3/3 trials)	100% (3/3 trials)	84.2%	67.7%	93.7%	83.3%	73.3%
Vignette 6. Blurting out offensive language obsessions	100% (1/1 trial)	100% (1/1 trial)	100% (1/1 trial)	N/A	26.1%	74.5%	N/A	N/A
Vignette 7. Somatic obsessions	100% (1/1 trial)	0% (0/1 trial)	100% (1/1 trial)	N/A	60.0%	82.4%	N/A	N/A
Vignette 8. Symmetry obsessions	100% (2/2 trials)	100% (2/2 trials)	100% (2/2 trials)	N/A	96.3%	100.0%	85.0%	71.4%

^a^The top five degrees/licenses of the American Psychological Association (APA) members were PhD (67.6%), MA/MS (31.5%), PsyD (14.2%), EdD/EdS/EdM (6.8%), MSW/LMSW (1.7%). Currently licensed was 81.3%.

^b^The areas of specialty included Internal Medicine (35.4%), Pediatrics (32.3%), Family Medicine (22.2%), other specialties (10.6%), and General Medicine (4.5%).

^c^The degrees include Clinical Psychology with Health Emphasis PhD, School-Clinical PsyD, School-Clinical PhD, Clinical Psychology PsyD, and Clinical Psychology PhD in 7 APA-accredited doctoral programs in the Greater New York area.

^d^Group 4 includes medical doctors, nurse practitioners, physician assistants, and doctors of Osteopathic Medicine. The areas of specialty included Internal Medicine (33.3%), Family Medicine (26.7%), Pediatrics (16.2%), Obstetrics and Gynecology (6.7%), Emergency Medicine (4.8%), and other (12.6%).

^e^The sample size differs between LLMs and mental health and health care professionals due to study design (LLM: responses from three LLM models; Human participants: responses from the wide distribution of the vignette studies).

^f^LLM (ChatGPT-4) did not respond to all three vignette trials due to content violation.

^g^LLM (Gemini Pro) did not respond to one vignette trial due to content violation.

^h^LLM (Gemini Pro) did not respond to two vignette trials due to content violation.

**Fig. 1:**
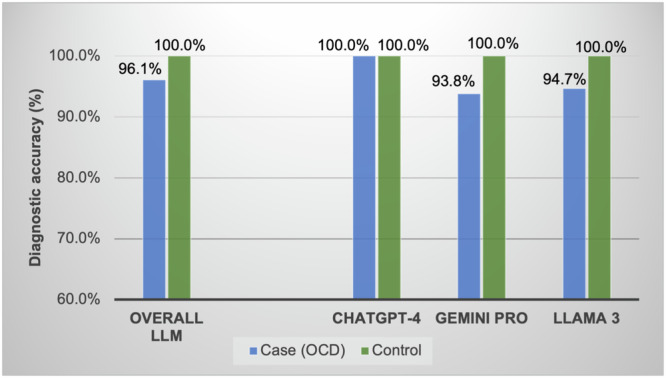
Diagnostic accuracy of LLMs by case (OCD) and control (other psychiatric disorders). Overall LLM performance: Case (*N* = 49/51) and control (*N* = 21/21). ChatGPT-4 and Gemini Pro (Case: 16 OCD vignettes) and Llama 3 (Case: 19 OCD vignettes). All LLMs had the same control group comprised of seven psychiatric disorders (major depressive disorder, generalized anxiety disorder, post-traumatic stress disorder, uni or bipolar depression, depression among adolescents, social anxiety disorder, and panic disorder).

Our results show that the LLMs, deployed in a zero-shot approach, were consistently more accurate in identifying OCD vignettes compared to mental health and medical professionals, doctoral students, and clergy members. ChatGPT-4’s perfect diagnostic performance with accurate clinical reasoning in diagnosing OCD was notable. Results could be improved if we had prompted or finetuned the LLMs, suggesting the even greater potential of the use of LLMs in mental health diagnostic support. There is growing evidence that patients use LLMs for self-diagnosis and medical information and may feel more comfortable disclosing this information to a chatbot than a clinician^21,22^. These findings suggest that LLMs may be a viable tool for increasing the efficiency and accuracy of OCD diagnostic assessment, or at least serving as a valuable and easily accessible screening tool.

To the best of our knowledge, this is the first study that evaluated LLMs’ diagnostic performance and clinical reasoning in OCD. Despite the potential benefit of augmenting clinical care with LLMs, further research is needed to weigh ethical issues and risks/benefits of improved diagnostic accuracy^23^. While we observed a slight variation in diagnostic performance by LLM, remarkably, clinical reasoning was comprehensive for all correct cases. Further exploration is needed to understand precisely how and when these tools could be deployed to maximize outcomes within the workflow of clinical practice. An important limitation of our study is that it uses vignettes rather than clinical histories from electronic medical records; any tests of efficacy need to include real-world clinical data to further understand the rates of false positives and negatives and the challenges this may present. Another limitation was the LLMs’ inability to provide a response to an OCD vignette that contained specific sexual content, which violated the platform’s content rules. Although the LLM correctly identified OCD in the vignettes presented, it is unclear if this accuracy would also be consistent across a larger range of mental health diagnoses or OCD symptom presentations.

The public health benefit of expediting and improving the accuracy of clinicians’ ability to detect OCD symptoms is the impact it would have on individuals early in the course of illness, before it severely disrupts school, work, relationships, and quality of life. Further studies are warranted to assess the effectiveness of using LLMs to aid with diagnostic assessments and treatment suggestions for psychiatric disorders within mental health and primary settings. Well-designed clinical studies can generate practical knowledge in the applications of LLMs in psychiatry, which might help in the wise adoption of these technologies in this area of medicine. Given the potential public health benefits of earlier diagnosis and improved care for patients with OCD, this use of LLM merits further testing and consideration.

## Methods

We identified five prior studies that assessed providers’ ability to identify OCD using clinical vignettes from mental health providers, medical doctors, psychology doctoral students, and clergy members (see Supplementary Table 1). We applied a case-control design to mitigate undetected potential biases—including selection bias—varying the sources of a control group for LLM performance. We used a 1:1 ratio of clinical vignettes of OCD as a case and other psychiatric disorders as a control group. We tested a total of 19 OCD vignettes, presenting 8 different OCD types, from five original previously published studies^24–28^. Seven control vignettes included major depressive disorder, generalized anxiety disorder, post-traumatic stress disorder, uni or bipolar depression, depression among adolescents, social anxiety disorder, and panic disorder from seven validated sources. For control vignettes, a list of suggested options for disorders was not provided except for one vignette, as we intended to adapt the validated vignettes (see Supplementary Table 2).

We assessed the performance of three different AI chatbots to reduce any potential biases that may come from a specific LLM^29^. Three AI chatbots included ChatGPT-4 (Open AI, Inc., April 2023 version), Gemini Pro (Google Inc., April 2024 version), and Llama 3 (Meta Inc., April 2024 version). We used a zero-shot approach in that we did not prompt or finetune the chatbots with the goal of improving their performance^30^. The three AI chatbots were asked to provide the three most likely medical diagnoses, rank their choice in order of likelihood, and offer clinical reasoning behind their diagnoses^31^. Each vignette was input into a fresh session of the chatbots, and the first response was recorded. Primary diagnosis, which was ranked first, was counted as the correct identification of the disorder for OCD cases and all controls. The correct OCD identification rates in three AI chatbots were compared with medical and mental health professionals’ performance assessed using the same OCD vignettes in the five original studies. Diagnostic performance for control vignettes was examined across three AI chatbots. Lastly, two mental health professionals independently reviewed the AI chatbots’ responses to detect any incorrect clinical reasoning behind their diagnoses. Inter-rater agreement was calculated as a Cohen’s kappa. The present study involved no personally identifiable human subject information; hence, it was deemed exempt from the Institutional Review Board at Stanford University.

### Supplementary information

Supplemental Material

## Data Availability

The vignettes used in this study were publicly available from published literature, and the vignette information is provided in a footnote of Supplementary Table 3. The data generated from this study, including OCD and control vignettes and LLMs’ responses to them, will be made available through Supplemental Material upon publication of this study.
