# Supplementary material for: Large language models outperform mental and medical health care professionals in identifying obsessive-compulsive disorder
Source: NPJ Digit Med. 2024 Jul 19;7:193. doi: 10.1038/s41746-024-01181-x (PMC11271579; doi:10.1038/s41746-024-01181-x)
Supplement: Supplementary file 1 — Supplemental Material [file 41746_2024_1181_MOESM1_ESM.pdf]

Supplemental Material

Title: Large language models outperform mental and medical health care professionals in identifying obsessive-compulsive disorder

- Supplementary Table 1. Demographic characteristics of comparison groups
- Supplementary Table 2. Large language models' (LLMs) diagnoses for control vignettes
- Supplementary Table 3. Large language models' (LLMs) primary and secondary diagnoses for OCD vignettes
- LLM responses and reasoning for OCD cases
- Control vignettes and LLMs responses

Supplementary Table 1. Demographic characteristics of comparison groups

	Group 1	Group 2	Group 3	Group 4	Group 5
Participants	Mental health professionals (American Psychological Association members)	Primary care physicians (Greater New York area)	Doctoral trainees in psychology	Non-psychiatry medical doctors in Guam[a]	Clergy in Guam[b]
Number of participants (*N*)	360	208	82	105	110
Year of participation	2011	2012-2013	2013	2019	2019
Age (mean, [*SD*] in years)	51.8 [12.6]	46.8 [11.4]	27.0 [3.8]	47.5 [12.7]	50.0 [13.1]
**Gender (%)**					
Female	57.5	57.2	82.6	40.0	30.0
Male	42.5	42.8	17.4	60.0	70.0
**Race/Ethnicity (%)**					
Asian	1.7	14.5	9.9	34.3	31.6
Black/ African American	2.0	8.8	3.7	1.00	4.3
White/ Caucasian	84.4	76.7	84.0	44.8	35.0
Other	2.7	3.6	2.5	22.0	29.0

[a] They were recruited through the Guam Medical Association, Guam Medical Society, Guam Memorial Hospital, Guam Regional Medical Center, and local clinics.
[b] Religious affiliation included Baptist (30.9%), Non-denominational (18.2%), Pentecostal (15.5%), Catholic (13.6%), Seventh-Day Adventist (10.0%), Evangelical (9.1%), and other (2.7%).

Supplementary Table 2. Large language models' (LLMs) diagnoses for control[a] vignettes[b]

	ChatGPT-4	Gemini Pro	Llama 3
Control vignette 1.<br>Major depressive disorder (MDD)[1]	1.Postpartum depression (PPD)<br>2.Generalized Anxiety Disorder (GAD)<br>3.Thyroids dysfunction	1. PPD<br>2. GAD<br>3. Adjustment disorder with mixed anxiety and depressed mood	1. PPD<br>2. GAD or Adjustment disorder with anxiety<br>3. Iron deficiency anemia
Control vignette 2.<br>Generalized anxiety disorder (GAD)[2]	1. **GAD**<br>2. MDD<br>3. Chronic stress	1. **GAD**<br>2. Adjustment disorder with mixed anxiety and depressed mood<br>3. Post-traumatic stress disorder (PTSD)	1. **GAD**<br>2. Adjustment disorder with anxiety<br>3. Depression
Control vignette 3.<br>Post-traumatic stress disorder (PTSD)[3]	1. **PTSD**<br>2. MDD<br>3. Anxiety disorder	1. **PTSD**<br>2. MDD<br>3. GAD	**1. PTSD**<br>2. Anxiety disorder<br>3. MDD
Control vignette 4.<br>MDD[4]	1. **MDD**<br>2. GAD<br>3. Bipolar disorder	1. **MDD**<br>2. GAD<br>3. Adjustment disorder with mixed anxiety and depressed mood	1. **MDD**<br>2. Anxiety disorder<br>3. Obstructive sleep apnea
Control vignette 5.<br>Uni or bipolar depression[5]	1. **Unipolar MDD**<br>2. GAD<br>3. Depression as a part of bipolar disorder<br>4. Substance-induced mood disorder<br>5. Attention-deficit hyperactivity disorder (ADHD)	1. **Unipolar MDD**<br>2. **Depression as a part of bipolar disorder**<br>3. Substance-induced mood disorder<br>4. GAD<br>5. ADHD	1. **Unipolar MDD**<br>2. **Depression as a part of bipolar disorder**<br>3. GAD<br>4. ADHD<br>5. Substance-induced mood disorder
Control vignette 6.<br>Social anxiety disorder[6]	1**. Social anxiety disorder (SAD)**<br>2. GAD<br>3. Panic disorder	1. **SAD**<br>2. Specific phobia<br>3. GAD	1**. SAD**<br>2. Panic disorder<br>3. GAD
Control vignette 7.<br>Panic disorder[7]	1. **Panic disorder**<br>2. Agoraphobia<br>3. GAD	1. **Panic disorder**<br>2. GAD<br>3. SAD	1. **Panic disorder**<br>2. GAD or SAD<br>3. Cardiac arrhythmia or other cardiac condition

[a] For control vignettes, a list of psychiatric disorders to choose from was not provided, except for control vignette 5.

[b] Control vignettes sources:

1. Price D. Evidence-Based Clinical Vignettes from the Care Management Institute: Major Depression. *Perm J*. 2002;6(1):34-42. Accessed March 12, 2024. https://www.ncbi.nlm.nih.gov/pmc/articles/PMC6220621/

2. *Generalised Anxiety Disorder and Panic Disorder in Adults: Management*. National Institute for Health and Care Excellence (NICE); 2019. Accessed March 12, 2024. http://www.ncbi.nlm.nih.gov/books/NBK552847/

3. Victor (post-traumatic stress disorder) | Society of Clinical Psychology. Accessed March 12, 2024. https://div12.org/case_study/victor-ptsd/

4. Clinical case study: CBT for depression in a Puerto Rican adolescent: challenges and variability in treatment response - Jiménez Chafey - 2009 - Depression and Anxiety - Wiley Online Library. Accessed March 12, 2024. https://onlinelibrary.wiley.com/doi/10.1002/da.20457

5. Cook IA. Recurrent Major Depressive Disorder of a Young Woman. *FOCUS*. 2016;14(2):210-213. doi:10.1176/appi.focus.20150047

6. Leichsenring F, Leweke F. Social anxiety disorder. *N Engl J Med*. 2017;376(23):2255-2264. doi:10.1056/NEJMcp1614701

7. Dave (panic disorder) | Society of Clinical Psychology. Published December 22, 2018. Accessed March 12, 2024. https://div12.org/case_study/dave-panic-disorder/

Supplementary Table 3. Large language models' (LLMs) primary and secondary diagnoses for OCD vignettes

	ChatGPT-4			Llama 3 (April 2024)			Gemini Pro (April 2024)		
	2013 vignettes[a]	2015 vignettes[b]	2022 vignettes[c]	2013 vignettes[a]	2015 vignettes[b]	2022 vignettes[c]	2013 vignettes[a]	2015 vignettes[b]	2022 vignettes[c]
Vignette 1. Harm obsessions	1. OCD	1. OCD	1. OCD	1. OCD<br>2.GAD<br>3. Social phobia/social anxiety disorder	1. OCD<br>2. GAD	1. OCD<br>2. GAD<br>3. Panic disorder	1. OCD<br>2. GAD<br>3. Specific phobia (harm OCD type)	1. OCD<br>2. GAD<br>3. BDD (Body dysmorphic disorder)	1. OCD<br>2. Social phobia/social anxiety disorder<br>3. GAD<br>4. Impulse control disorder
Vignette 2. Sexual orientation obsessions	1. OCD	1. OCD	1. OCD	1. Sexual identity confusion<br>2. Social phobia/social anxiety disorder	1. OCD<br>2. GAD	1. OCD<br>2. GAD	No response: content violation	1. OCD<br>2. Sexual identity confusion<br>3. BDD (Body dysmorphic disorder)	1. OCD<br>2. Social phobia/social anxiety disorder<br>3. GAD<br>4. Impulse control disorder
Vignette 3. Sexual attraction to children obsessions	No response: content violation	No response: content violation	No response: content violation	1. OCD	1. OCD<br>2. GAD	1. OCD<br>2. GAD	1. OCD	No response: content violation	No response: content violation
Vignette 4. Religious obsessions	1. OCD	1. OCD	1. OCD	1. OCD<br>2. Specific phobia	1. OCD<br>2. GAD	1. OCD<br>2. GAD	1. OCD<br>2. GAD<br>3. Specific phobia	1. OCD<br>2. GAD	1. OCD<br>2. GAD

Vignette 5. Contamination obsessions	1. OCD	1. OCD	1. OCD	1. OCD<br>2. GAD<br>3. Social phobia/social anxiety	1. OCD<br>2. GAD<br>3. Hypochondriasis	1. OCD<br>2. GAD<br>3. OCPD	1. OCD<br>2. Specific phobia<br>3. GAD	1. OCD<br>2. GAD<br>3. Specific phobia<br>4. Hypochondriasis	1. OCD<br>2. Specific phobia<br>3. Hypochondriasis<br>4. SAD
Vignette 6. Blurting out offensive language obsessions	N/A	1. OCD	N/A	N/A	1. OCD<br>2. Social phobia/social anxiety disorder<br>3. GAD	N/A	N/A	1. OCD<br>2. GAD<br>3. Social Anxiety Disorder (SAD)	N/A
Vignette 7. Somatic obsessions	N/A	1. OCD<br>2. GAD	N/A	N/A	1. OCD<br>2. BDD (Body dysmorphic disorder)<br>3. GAD	N/A	N/A	1. BDD<br>2. SAD	N/A
Vignette 8. Symmetry obsessions	N/A	1. OCD<br>2. OCPD	1. OCD<br>2. OCPD	N/A	1. OCD<br>2. OCPD	1. OCD<br>2. OCPD	N/A	1. OCD<br>2. OCPD<br>3. GAD<br>4. ASD (Autism Spectrum Disorder)	1. OCD<br>2. OCPD<br>3. BDD

[a] 2013 version included 5 OCD vignettes with a list of 36 example psychiatric disorders. This was tested for mental health professionals (American Psychology Association members)
[b] 2015 version included 8 OCD vignettes with a list of 47 example psychiatric disorders. This was tested for primary care physicians and doctoral trainees in Psychology.
[c] 2022 version included 6 OCD vignettes with a list of 44 example psychiatric disorders. This was tested for non-psychiatry healthcare professionals and clergy in Guam.
Abbreviations: OCD (Obsessive compulsive disorder), OCPD (Obsessive compulsive personality disorder), GAD (Generalized anxiety disorder), MDD (Major depressive disorder)

1. LLM responses and reasoning for OCD cases

2013 version for APA members

Vignette 1 (2013)

Jack, a middle-aged man, thought about pushing the lady next to him onto the subway tracks. He was distressed by the thought and the fear that he might act on it, so he left the subway and walked home. However, Jack remained worried and found himself frequently visualizing the situation to make sure he did not actually cause her any harm. This worry about harming others occurs often and causes Jack significant distress.

Based on the vignette which option most likely applies? If more than one, please mark all options that may apply. If you marked more than one of the above, please rank your choices in order of likelihood.
Lastly, use behavioral signs and symptoms from the vignette to step by step deduce the correct response and show me your reasoning for your choice of most likely option.

1. Agoraphobia with panic disorder
2. Agoraphobia without panic disorder
3. Anger management issues
4. Anorexia
5. Antisocial personality disorder
6. Attention deficit hyperactivity disorder
7. Autism
8. Avoidant personality disorder
9. Bipolar I disorder
10. Bipolar II disorder
11. Borderline personality disorder
12. Bulimia
13. Delirium
14. Delusional disorder
15. Generalized anxiety disorder
16. Histrionic personality disorder
17. Impulse control disorder
18. Intermittent explosive disorder
19. Major depression
20. Marital problems
21. Narcissistic personality disorder
22. Obsessive-compulsive disorder
23. Obsessive-compulsive personality disorder
24. Organic disorder
25. Panic disorder
26. Paranoid personality disorder
27. Posttraumatic stress disorder
28. Pedophilia
29. Psychosis
30. Sexual identity confusion

31. Schizophrenia
32. Schizotypal personality disorder
33. Schizoid personality disorder
34. Social phobia/Social anxiety disorder
35. Specific phobia
36. Strong religious values

LLM responses to vignette 1.

V1 (2013). ChatGPT-4

In the provided vignette, Jack is experiencing significant distress due to doubts about his sexual orientation, despite being in a committed heterosexual relationship and not having a history of sexual attraction to males. He often questions his sexuality and assesses his sexual arousal when seeing males, which suggests the presence of intrusive thoughts and the performance of mental acts in response (i.e., checking).

The option that most likely applies based on the behavioral signs and symptoms described is:

22. Obsessive-compulsive disorder (OCD)

The reasoning for this choice is as follows:

- **Intrusive Thoughts**: Jack has recurring doubts about his sexuality, which are intrusive and cause him significant distress.
- **Checking Behavior**: Jack feels compelled to check his sexual arousal when seeing males, which can be seen as a compulsion in response to his intrusive thoughts.
- **Distress**: The doubt and checking cause Jack significant distress, which is a key feature of OCD where obsessions and compulsions cause marked distress or significant impairment.
- **No Indication of Sexual Attraction to Males**: Despite his checks, Jack has never been sexually attracted to males, which suggests that the distress is more about the uncertainty and the need for reassurance rather than a genuine questioning of sexual orientation.

None of the other disorders listed in the options provided (such as Agoraphobia, Anger management issues, Eating disorders, Personality disorders, ADHD, Autism, Mood disorders, Delirium, Delusional disorder, Impulse control disorders, Marital problems, Organic disorder, PTSD, Psychosis, Schizophrenia, Specific phobias, or Strong religious values) describe a pattern of intrusive, unwanted thoughts paired with checking behaviors and significant distress related to the content of the obsessions.

As for Sexual identity confusion (Option 30), while this could be considered, it is less likely in the absence of a genuine questioning of sexual orientation or desire for same-sex experiences. Instead, Jack's pattern of behavior is more in line with OCD, where the content of the obsessions can often focus on distressing doubts about one's identity, including sexual orientation.

V1 (2013). Llama 3

Based on the vignette, I believe the most likely option is:
22. Obsessive-compulsive disorder (OCD)

Here's my step-by-step reasoning:
1. The vignette mentions that Jack has a distressing thought about pushing a lady onto the subway tracks, which suggests an intrusive and unwanted thought.

2. Jack is distressed by this thought and fears that he might act on it, which indicates anxiety and apprehension.

3. To cope with this anxiety, Jack leaves the subway and walks home, which may be an attempt to avoid the situation and reduce his anxiety.

4. Despite leaving the situation, Jack continues to worry and frequently visualizes the scenario to ensure he didn't harm anyone. This repetitive mental act is a common characteristic of OCD.

5. The vignette states that this type of worry about harming others occurs often and causes Jack significant distress, which suggests a pattern of behavior consistent with OCD.

Other options that could potentially apply, although less likely, are:
15. Generalized anxiety disorder (GAD) - Jack's excessive worry and anxiety could be indicative of GAD. However, the presence of intrusive thoughts and compulsive mental acts (visualizing the scenario) points more strongly towards OCD.
34. Social phobia/Social anxiety disorder - Jack's avoidance of the subway situation could be seen as avoidance of social situations due to fear of negative evaluation or embarrassment.

However, the primary focus on intrusive thoughts and compulsive behaviors makes OCD a more likely diagnosis.

Ranking my choices in order of likelihood:
1. Obsessive-compulsive disorder (OCD)
2. Generalized anxiety disorder (GAD)
3. Social phobia/Social anxiety disorder

V1 (2013). Gemini pro

Determining the Most Likely Diagnosis
Based on the information provided, the most likely diagnoses for Jack's situation are:

1. Obsessive-compulsive disorder (OCD) with intrusive thoughts: This is the most likely diagnosis as Jack experiences recurrent intrusive thoughts about harming others, causing significant distress and anxiety. He engages in mental compulsions (visualizations) to try and prevent the feared outcome, which is a hallmark of OCD.

2. Generalized anxiety disorder (GAD): Jack's frequent worrying, even in the absence of immediate danger, aligns with GAD. His distress and inability to control his thoughts contribute to this diagnosis.

3. Specific phobia - Harm OCD subtype: Although less likely than full-blown OCD, Jack's fear of harming others could be classified as a specific phobia with an OCD component. This diagnosis would focus specifically on the fear of causing harm and the associated anxiety, while acknowledging the intrusive thoughts and compulsive behaviors.

Likelihood Ranking:

1. Obsessive-compulsive disorder (OCD) with intrusive thoughts
2. Generalized anxiety disorder (GAD)
3. Specific phobia - Harm OCD subtype

Reasoning and Behavioral Signs

OCD:

- Intrusive thoughts: Jack has recurrent and unwanted thoughts about pushing the lady onto the subway tracks.
- Anxiety and distress: These thoughts cause him significant anxiety and fear.
- Mental compulsions: He visualizes the situation to prevent himself from acting on his intrusive thoughts.
- Lack of pleasure in the thoughts: Jack does not find these thoughts enjoyable or desirable.

GAD:

- Excessive worry: Jack frequently worries about various things, including harming others.
- Difficulty controlling worry: He finds it difficult to control his worrying thoughts.
- Restlessness and fatigue: His anxiety may manifest as physical symptoms like restlessness and fatigue.

Specific phobia - Harm OCD subtype:

- Intense fear of causing harm: This is the core feature of this diagnosis.
- Avoidance of situations: Jack may avoid situations where he fears he might harm someone.
- Physical symptoms of anxiety: He may experience physical symptoms like sweating, racing heart, and nausea when experiencing the fear.

Important Note

This analysis is based on limited information and should not be considered a definitive diagnosis. A qualified mental health professional should conduct a comprehensive evaluation to determine the most accurate diagnosis and recommend the appropriate treatment plan.

Vignette 2 (2013)

Jack, a middle-aged man, has been in a committed relationship with his girlfriend for 2 years. While he loves his girlfriend and has never been sexually attracted to males, he finds himself wondering if he really is gay. Upon seeing males, Jack assesses his sexual arousal to

determine if he is turned on. This doubt regarding his sexuality occurs often and causes Jack significant distress.

Based on the vignette which option most likely applies? If more than one, please mark all options that may apply. If you marked more than one of the above, please rank your choices in order of likelihood.
Lastly, use behavioral signs and symptoms from the vignette to step by step deduce the correct response and show me your reasoning for your choice of most likely option.

1. Agoraphobia with panic disorder
2. Agoraphobia without panic disorder
3. Anger management issues
4. Anorexia
5. Antisocial personality disorder
6. Attention deficit hyperactivity disorder
7. Autism
8. Avoidant personality disorder
9. Bipolar I disorder
10. Bipolar II disorder
11. Borderline personality disorder
12. Bulimia
13. Delirium
14. Delusional disorder
15. Generalized anxiety disorder
16. Histrionic personality disorder
17. Impulse control disorder
18. Intermittent explosive disorder
19. Major depression
20. Marital problems
21. Narcissistic personality disorder
22. Obsessive-compulsive disorder
23. Obsessive-compulsive personality disorder
24. Organic disorder
25. Panic disorder
26. Paranoid personality disorder
27. Posttraumatic stress disorder
28. Pedophilia
29. Psychosis
30. Sexual identity confusion
31. Schizophrenia
32. Schizotypal personality disorder
33. Schizoid personality disorder
34. Social phobia/Social anxiety disorder
35. Specific phobia
36. Strong religious values

LLM responses to vignette 2.

V2 (2013). ChatGPT-4

The behavioral signs and symptoms described in the vignette suggest that Jack exhibits behaviors that align with obsessive-compulsive behaviors, particularly those involving religious or moral themes. Here is the step-by-step deduction:

1. **Religious Preoccupations**: Jack's beliefs and the distress he feels when those beliefs are challenged or when he perceives blasphemy indicate a strong preoccupation with religious matters.
2. **Compulsive Behaviors**: Jack responds to this distress by engaging in a repetitive behavior, specifically prayer, which he feels compelled to continue until he achieves a sense of safety.
3. **Distress and Functional Impairment**: The behavior of praying for hours suggests that it significantly impacts his daily functioning and causes him distress.
4. **Specific Trigger**: The compulsive behavior is triggered by specific events – hearing negative expressions about religious figures.

Based on these points, the most likely options are:

- **Obsessive-compulsive disorder (22)**: This disorder is characterized by the presence of obsessions (unwanted, intrusive thoughts that cause distress) and compulsions (repetitive behaviors that an individual feels driven to perform in response to an obsession or according to rules that must be applied rigidly).

Other options do not fit the behaviors described. For example, agoraphobia involves fear of places or situations that might cause panic or helplessness, which is not indicated here. Anger management issues involve difficulty in controlling anger, which Jack does not exhibit. Anorexia and bulimia are eating disorders, which are not mentioned. Antisocial personality disorder involves a disregard for others' rights, which is not evident in Jack's case.

None of the other disorders listed (e.g., ADHD, Autism, Bipolar disorders, Personality disorders, etc.) have features that align with Jack's symptoms as described. While strong religious values (36) are apparent, the degree of distress and the compulsion to pray for hours suggest a disorder rather than simply strong values.

Therefore, the most likely diagnosis is:

22. Obsessive-compulsive disorder

This choice is made based on the presence of both obsessions (excessive worries about blasphemy) and compulsions (repeated prayers) that are clearly interfering with Jack's functioning.

V2 (2013). Llama 3

Based on the vignette, the most likely option that applies is:
30. Sexual identity confusion

Here's my step-by-step reasoning:
1. The vignette states that Jack has been in a committed relationship with his girlfriend for 2 years and has never been sexually attracted to males. This suggests that Jack's primary sexual orientation is heterosexual.

2. However, Jack finds himself wondering if he is really gay, which indicates that he is experiencing some level of uncertainty or confusion about his sexual identity.

3. The fact that Jack assesses his sexual arousal when seeing males to determine if he is turned on implies that he is actively trying to figure out his sexual orientation. This behavior suggests that Jack is struggling with his sexual identity.

4. The vignette also mentions that this doubt regarding his sexuality occurs often and causes Jack significant distress. This emotional distress is a key indicator of sexual identity confusion.

Ranking my choices in order of likelihood:
1. 30. Sexual identity confusion (most likely)
2. 34. Social phobia/Social anxiety disorder (possible, as Jack may be experiencing anxiety related to his sexual identity confusion)I did not consider other options, such as anxiety disorders or personality disorders, as the primary focus of the vignette is on Jack's sexual identity confusion and its associated distress.

V2 (2013). Gemini pro

The response was blocked because the input or response may contain descriptions of violence, sexual themes or otherwise derogatory content. Please try rephrasing your prompt.

Vignette 3 (2013)

Jack, a middle-aged man, used to love spending time with his nieces and nephews and is their “favorite uncle.” Recently he had an image of touching one of the children inappropriately, which upset him greatly. He was certain that he’d never harm them but the thoughts increased in frequency. He now tries to avoid contact with the children and will not spend time alone with them. Jack’s fear of acting on these thoughts occurs often and causes him significant distress.

Based on the vignette which option most likely applies? If more than one, please mark all options that may apply. If you marked more than one of the above, please rank your choices in order of likelihood.
Lastly, use behavioral signs and symptoms from the vignette to step by step deduce the correct response and show me your reasoning for your choice of most likely option.

1. Agoraphobia with panic disorder
2. Agoraphobia without panic disorder

3. Anger management issues
4. Anorexia
5. Antisocial personality disorder
6. Attention deficit hyperactivity disorder
7. Autism
8. Avoidant personality disorder
9. Bipolar I disorder
10. Bipolar II disorder
11. Borderline personality disorder
12. Bulimia
13. Delirium
14. Delusional disorder
15. Generalized anxiety disorder
16. Histrionic personality disorder
17. Impulse control disorder
18. Intermittent explosive disorder
19. Major depression
20. Marital problems
21. Narcissistic personality disorder
22. Obsessive-compulsive disorder
23. Obsessive-compulsive personality disorder
24. Organic disorder
25. Panic disorder
26. Paranoid personality disorder
27. Posttraumatic stress disorder
28. Pedophilia
29. Psychosis
30. Sexual identity confusion
31. Schizophrenia
32. Schizotypal personality disorder
33. Schizoid personality disorder
34. Social phobia/Social anxiety disorder
35. Specific phobia
36. Strong religious values

LLM responses to vignette 3.

V3 (2013). ChatGPT-4: N/A

V3 (2013). Llama 3

Based on the vignette, I believe the most likely option is:
22. Obsessive-Compulsive Disorder (OCD)

Here's my step-by-step reasoning:
1. Jack experiences recurring, intrusive thoughts of touching one of his nieces or nephews inappropriately, which causes him significant distress. This is a classic symptom of obsessive thinking.

2. The frequency of these thoughts increases over time, which suggests a pattern of obsessive thinking.

3. Jack is certain that he would never harm his nieces and nephews, but the thoughts persist, causing him anxiety and fear. This indicates that he is experiencing ego-dystonic thoughts, which are thoughts that are unacceptable or repugnant to the individual.

4. As a result of these thoughts, Jack begins to avoid contact with his nieces and nephews and refuses to spend time alone with them. This avoidance behavior is a common coping mechanism for individuals with OCD who are trying to prevent perceived harm or danger.

5. The vignette does not mention any other symptoms or behaviors that would suggest alternative diagnoses, such as panic attacks (Agoraphobia), anger outbursts (Anger management issues), or disordered eating (Anorexia or Bulimia).

In ranking my choices, I would place Obsessive-Compulsive Disorder (OCD) as the most likely option, followed by:15. Generalized Anxiety Disorder (GAD) - Although Jack's symptoms are more specific to OCD, GAD could also be considered due to his excessive worry and anxiety about acting on his intrusive thoughts. I do not think the other options are likely based on the information provided in the vignette.

V3 (2013). Gemini pro

Applying Diagnostic Criteria to Jack's Situation:

Based on the vignette, Jack's situation most likely aligns with Obsessive-Compulsive Disorder (OCD), specifically with intrusive thoughts. Here's a step-by-step explanation:

1. Symptoms:

- Intrusive Thoughts: Jack experiences recurrent and distressing thoughts of touching the children inappropriately. These thoughts are unwanted and cause significant anxiety.
- Compulsions: While the vignette doesn't explicitly mention compulsions, Jack's avoidance behavior (limiting contact and refusing to be alone with the children) can be seen as a form of compulsion. He feels driven to perform these actions to reduce the anxiety caused by his intrusive thoughts.
- Distress and Impairment: Jack's fear of acting on the intrusive thoughts causes him significant distress and interferes with his life, affecting his relationships with his nieces and nephews.

2. Ruling out other options:

- Disorders related to social anxiety: While Jack may experience some anxiety in social situations involving children, his primary distress stems from the intrusive thoughts and not a general fear of social interactions.
- Mood disorders: The vignette doesn't provide evidence of persistent low mood, hopelessness, or manic episodes that would suggest bipolar disorder or major depression.
- Trauma-related disorders: There's no indication in the vignette of a traumatic experience that could explain his symptoms.

- Personality disorders: Jack shows no consistent pattern of disregard for the rights of others, significant impulsivity, or unstable moods, ruling out options like antisocial personality disorder or borderline personality disorder.
- Other disorders: The remaining options listed don't align with the specific symptoms described in the vignette.

3. Supporting evidence for OCD: Studies show that individuals struggling with OCD commonly experience disturbing and unwanted thoughts of a sexual nature, causing significant distress. Therefore, based on the information provided, Obsessive-Compulsive Disorder is the most likely diagnosis. However, it's crucial to remember that this is not a definitive diagnosis and only a qualified mental health professional can provide an accurate diagnosis after a comprehensive assessment.

Vignette 4 (2013)

Jack, a middle-aged, highly religious man, believes that he is not allowed to say the Lord's name in vain. Moreover, if he hears someone else say anything remotely negative toward God, Jesus, or the Virgin Mary, he feels significant distress, fearing for his and the individual's safety and well-being. Therefore, upon hearing such expressions, Jack prays repeatedly to himself until he feels safe from harm. This can go on for hours.

Based on the vignette which option most likely applies? If more than one, please mark all options that may apply. If you marked more than one of the above, please rank your choices in order of likelihood.
Lastly, use behavioral signs and symptoms from the vignette to step by step deduce the correct response and show me your reasoning for your choice of most likely option.

1. Agoraphobia with panic disorder
2. Agoraphobia without panic disorder
3. Anger management issues
4. Anorexia
5. Antisocial personality disorder
6. Attention deficit hyperactivity disorder
7. Autism
8. Avoidant personality disorder
9. Bipolar I disorder
10. Bipolar II disorder
11. Borderline personality disorder
12. Bulimia
13. Delirium
14. Delusional disorder
15. Generalized anxiety disorder
16. Histrionic personality disorder
17. Impulse control disorder
18. Intermittent explosive disorder
19. Major depression
20. Marital problems
21. Narcissistic personality disorder
22. Obsessive-compulsive disorder
23. Obsessive-compulsive personality disorder

24. Organic disorder
25. Panic disorder
26. Paranoid personality disorder
27. Posttraumatic stress disorder
28. Pedophilia
29. Psychosis
30. Sexual identity confusion
31. Schizophrenia
32. Schizotypal personality disorder
33. Schizoid personality disorder
34. Social phobia/Social anxiety disorder
35. Specific phobia
36. Strong religious values

LLM responses to vignette 4.

V4 (2013). ChatGPT-4

Based on the description provided, here are the steps to deduce the most likely option:

- Jack experiences significant distress when he perceives negative remarks towards religious figures, which leads to a compulsive behavior (praying repeatedly).
- The behavior is a ritual performed to prevent or reduce distress or prevent some dreaded event or situation; this is not related to a realistic threat.
- The compulsive praying continues until he feels safe, which can take hours, indicating that the behavior significantly impacts his daily life.

Considering the behavioral signs and symptoms:

1. There is no mention of fear of open spaces or panic attacks, ruling out agoraphobia with and without panic disorder.
2. Anger management issues do not seem to be present, as distress rather than anger is described.
3. There is no information suggesting anorexia.
4. Antisocial personality disorder is not indicated, as this would involve a disregard for others, which is not described.
5. ADHD is characterized by inattention, hyperactivity, and impulsivity, which is not described in the vignette.
6. Autism spectrum disorders are characterized by challenges with social skills and communication, which are not mentioned.
7. Avoidant personality disorder includes symptoms of social inhibition and feelings of inadequacy, which are not detailed here.
8. Bipolar disorders involve episodes of mood swings ranging from depressive lows to manic highs, which are not described.
9. Borderline personality disorder includes a pattern of unstable relationships, self-image, and affects, with impulsivity, not indicated in the vignette.
10. No symptoms of eating disorders such as bulimia are mentioned.

11. Delirium typically involves an acute onset and fluctuating course of confusion, which is not indicated.
12. Delusional disorder involves a presence of delusions, which is not clearly indicated.
13. Generalized anxiety disorder involves persistent and excessive worry about various things, not compulsive behaviors.
14. Histrionic personality disorder includes a pattern of excessive attention-seeking behaviors, not present here.
15. Impulse control disorder is marked by difficulties in controlling behaviors, not compulsive ritualistic behavior.
16. Intermittent explosive disorder is characterized by sudden episodes of unwarranted anger, not described here.
17. Major depression involves a persistent feeling of sadness and a lack of interest, which is not clearly described.
18. Marital problems are not mentioned in the vignette.
19. Narcissistic personality disorder includes a pattern of grandiosity, need for admiration, and a lack of empathy, not indicated here.
20. Obsessive-compulsive disorder (OCD) involves unwanted and intrusive thoughts (obsessions) and repetitive behaviors (compulsions) like what is described.
21. Obsessive-compulsive personality disorder includes a preoccupation with orderliness and perfectionism, not the distress over religious blasphemy.
22. Organic disorder would involve symptoms resulting from brain damage, which is not indicated.
23. Panic disorder is marked by sudden and repeated panic attacks, not described.
24. Paranoid personality disorder involves pervasive distrust and suspicion of others, not indicated.
25. PTSD is typically associated with a past traumatic event, which is not mentioned.
26. Pedophilia involves sexual attraction to children, which is not relevant here.
27. Psychosis involves a disconnection from reality, not indicated here.
28. Sexual identity confusion is not described in the vignette.
29. Schizophrenia involves symptoms like hallucinations or delusions, which are not clearly indicated.
30. Schizotypal and schizoid personality disorders include severe social anxiety, paranoia, and unconventional beliefs, not compulsive religious behaviors.
31. Social phobia/Social anxiety disorder involves an intense fear of social situations, not compulsive behavior following religious concerns.
32. Specific phobia involves an intense irrational fear of specific objects/situations, which is not the focus here.
33. There is no indication that Jack's religious values alone, without associated distress and compulsions, are the only concern.

Based on this analysis, the most likely diagnosis would be:

22. Obsessive-compulsive disorder (OCD) - Jack's compulsive need to pray in response to religious blasphemy and the significant distress associated with it, along with the impact on his daily life, align with the diagnostic criteria for OCD.

While "strong religious values" is listed, it does not qualify as a disorder unless it leads to significant distress or impairment, which in Jack's case, it does in the form of OCD.

The ranking of the most likely option is therefore:

1. Obsessive-compulsive disorder (22)
2. Strong religious values (36) - This could be a contributing factor but does not constitute a disorder on its own.

No other conditions in the list match the behavioral signs and symptoms described in the vignette as closely as OCD does.

V4 (2013). Llama 3

🦙
Based on the vignette, I believe the most likely options are:
1. **Obsessive-Compulsive Disorder (OCD)**
2. **Specific Phobia**

Here's my step-by-step reasoning:
1. Jack has a specific belief that he is not allowed to say the Lord's name in vain, and if he hears someone else do so, he experiences significant distress.

2. This distress is accompanied by a compulsive behavior, which is praying repeatedly to himself until he feels safe from harm.

3. The praying behavior can last for hours, indicating a sense of ritualistic or repetitive behavior, which is a hallmark of OCD.

4. Jack's fear of harm to himself and others suggests an underlying anxiety or phobic response.

5. The specific trigger for this response is hearing blasphemy or negative comments towards religious figures, which is a specific stimulus that elicits a predictable response.

Given these points, I believe OCD is the most likely option, as it explains Jack's intrusive thoughts, compulsive behaviors, and anxiety responses.

The second option, Specific Phobia, is also possible because Jack's response is triggered by a specific stimulus (hearing blasphemy), and his reaction involves significant anxiety and avoidance behaviors (praying to feel safe). However, the presence of compulsive behaviors (praying) makes OCD a more likely diagnosis. I ranked OCD as the top choice because it better explains the full range of symptoms described in the vignette, including the compulsive behaviors and anxiety responses.

V4 (2013). Gemini pro

Analyzing the vignette:

Behavioral signs and symptoms:
- Religious beliefs: Jack holds strong religious beliefs that prohibit him from saying the Lord's name in vain.

- Distress: He experiences significant distress when hearing negative comments about God, Jesus, or the Virgin Mary.
- Fear: He fears for his and the individual's safety and well-being.
- Compulsive behavior: He engages in repetitive praying for hours to feel safe.

Applying the signs and symptoms to potential diagnoses:

1. Obsessive-compulsive disorder (OCD): This seems like the most likely diagnosis. Jack's fear of saying the Lord's name in vain and his need to engage in repetitive praying to alleviate his distress are classic symptoms of OCD. His fear of negative comments about religious figures could also be related to intrusive thoughts, a common feature of OCD.

2. Religious Scrupulosity: This is a form of OCD that focuses on religious and moral obsessions. Jack's fear of blasphemy and the need to constantly seek reassurance through prayer are characteristics of religious scrupulosity.

3. Generalized anxiety disorder (GAD): Although GAD involves excessive worry, it usually focuses on various aspects of life, not specifically on religious matters. However, Jack's fear and distress could be considered excessive and contribute to a GAD diagnosis.

4. Specific phobia: While Jack's fear of blasphemy could be seen as a specific phobia, the broader range of his anxieties suggests a more complex issue than a singular phobia.

5. Delusional disorder: Although Jack's distress is significant, he doesn't appear to hold fixed false beliefs that are characteristic of delusional disorder.

Other options: The remaining options don't seem to fit the presented symptoms as well as the above-mentioned possibilities.

Ranking the most likely diagnoses:

1. Obsessive-compulsive disorder (OCD), with a possible focus on religious scrupulosity.
2. Generalized anxiety disorder (GAD), contributing to Jack's overall distress.
3. Specific phobia, specifically related to blasphemy, but likely not the sole cause of his anxieties.

Conclusion:

Based on the behavioral signs and symptoms described in the vignette, Jack most likely suffers from Obsessive-compulsive disorder (OCD), possibly with a religious scrupulosity focus. Generalized anxiety disorder and specific phobia might also contribute to his overall distress, but OCD seems to be the primary diagnosis due to the repetitive praying and fear of blasphemy.

Vignette 5 (2013)

Jack, a middle-aged man, constantly worries about dirt and germs. These worries limit his range of daily activities, for Jack tries at all costs to avoid touching objects he believes may be contaminated. However, if Jack does come in contact with a “dirty” object, he immediately

washes his hands to prevent himself from catching a disease. These worries occur often and cause Jack significant distress.

Based on the vignette which option most likely applies? If more than one, please mark all options that may apply. If you marked more than one of the above, please rank your choices in order of likelihood.
Lastly, use behavioral signs and symptoms from the vignette to step by step deduce the correct response and show me your reasoning for your choice of most likely option.

1. Agoraphobia with panic disorder
2. Agoraphobia without panic disorder
3. Anger management issues
4. Anorexia
5. Antisocial personality disorder
6. Attention deficit hyperactivity disorder
7. Autism
8. Avoidant personality disorder
9. Bipolar I disorder
10. Bipolar II disorder
11. Borderline personality disorder
12. Bulimia
13. Delirium
14. Delusional disorder
15. Generalized anxiety disorder
16. Histrionic personality disorder
17. Impulse control disorder
18. Intermittent explosive disorder
19. Major depression
20. Marital problems
21. Narcissistic personality disorder
22. Obsessive-compulsive disorder
23. Obsessive-compulsive personality disorder
24. Organic disorder
25. Panic disorder
26. Paranoid personality disorder
27. Posttraumatic stress disorder
28. Pedophilia
29. Psychosis
30. Sexual identity confusion
31. Schizophrenia
32. Schizotypal personality disorder
33. Schizoid personality disorder
34. Social phobia/Social anxiety disorder
35. Specific phobia
36. Strong religious values

LLM responses to vignette 5.

V5 (2013). ChatGPT-4

The vignette describes Jack, a middle-aged man who has a persistent worry about dirt and germs, which leads to a compulsion to wash his hands whenever he touches what he perceives to be a contaminated object. This behavior causes him significant distress and impacts his daily activities.

This description aligns with Obsessive-Compulsive Disorder (OCD). OCD is characterized by the presence of obsessions (recurring, persistent, intrusive thoughts) about contamination, which lead to compulsions (repetitive behaviors) such as hand washing. These symptoms are meant to neutralize or decrease distress related to the obsessions or to prevent some dreaded event or situation; however, these behaviors are not connected in a realistic way with what they are designed to neutralize or prevent, or are clearly excessive.

Here's the step by step deduction based on the vignette:

1. Jack has a persistent worry about dirt and germs — this points towards an anxiety-related issue, specifically a fear of contamination, which is an obsession.
2. This worry limits his daily activities as he tries to avoid touching objects he believes may be contaminated — indicating that the fear is significant enough to impact his functioning, a key aspect of a disorder.
3. If Jack comes into contact with a “dirty” object, he feels compelled to wash his hands immediately to prevent disease — this is a ritualistic behavior, known as a compulsion, aimed at addressing the obsession.
4. These worries occur often and cause significant distress — the frequency and level of distress further support the presence of a disorder that affects daily living.

Given the information provided, the most likely diagnosis is Obsessive-compulsive disorder (22). None of the other options listed align as closely with the signs and symptoms described in the vignette. Disorders like Agoraphobia (1, 2), various personality disorders (5, 8, 11, 21, 26, 32, 33), and others listed do not typically present with the specific pattern of obsessions and compulsions related to contamination and cleaning described here.

Therefore, the correct and most likely choice is: 22. Obsessive-compulsive disorder

The reasoning for this choice is Jack's specific pattern of obsessional thinking about contamination and his compulsive hand-washing behavior in response to these thoughts, which is a classic presentation of OCD.

V5 (2013). Llama 3

Based on the vignette, I believe the most likely option is:
22. Obsessive-compulsive disorder (OCD)

Here's my step-by-step reasoning:
1. The vignette mentions that Jack has excessive worries about dirt and germs, which is a common obsession in OCD.

2. Jack's behaviors are driven by these worries, as he tries to avoid touching objects he believes may be contaminated. This avoidance behavior is a hallmark of OCD.

3. When Jack does come into contact with a "dirty" object, he immediately washes his hands to prevent himself from catching a disease. This compulsive behavior (excessive handwashing) is another classic symptom of OCD.

4. The vignette states that these worries occur often and cause Jack significant distress, which is consistent with the diagnostic criteria for OCD.

I ranked my choices in order of likelihood as follows:
1. 22. Obsessive-compulsive disorder (OCD)
2. 15. Generalized anxiety disorder (GAD) - Although Jack's worries about dirt and germs are specific, they may also contribute to a broader pattern of excessive worry characteristic of GAD.
3. 34. Social phobia/Social anxiety disorder - Jack's avoidance behaviors might also be related to social anxiety or fear of being judged by others, but this is less likely given the specific focus on dirt and germs.
The other options do not appear to be directly applicable to Jack's symptoms based on the vignette.

V5 (2013). Gemini pro

Analyzing the vignette:

Based on the information provided in the vignette, Jack's behavior aligns most closely with Obsessive-compulsive disorder (OCD). Let's break down the reasoning step by step:

1. Behavioral signs and symptoms:
- Excessive worry about dirt and germs: This is a classic symptom of OCD, often manifesting as intrusive thoughts and anxieties.
- Avoidance of "dirty" objects: Jack's efforts to prevent contact with objects he perceives as contaminated directly reflect the avoidance compulsions associated with OCD.
- Washing hands immediately after contact with "dirty" objects: This compulsive behavior aims to alleviate the anxiety caused by the perceived contamination.
- Significant distress caused by these worries: The vignette explicitly states that Jack's anxieties considerably impact his daily life, further suggesting OCD.

2. Ruling out other options:
While some other disorders share some features with Jack's experience, they don't fully align with the presented scenario. For instance, specific phobias involve intense fear of specific objects or situations, but Jack's anxieties seem broader, encompassing a range of "dirty" objects. Additionally, although anxiety disorders like generalized anxiety disorder can involve excessive worry, they don't typically manifest in the specific compulsions observed in Jack's case.

Therefore, considering the specific anxieties, compulsions, and distress described, OCD emerges as the most likely diagnosis.

Ranking other potential options:
While OCD is the most likely diagnosis based on the information provided, other options could potentially apply with additional information. Here's a tentative ranking based on the available details:

1. Obsessive-compulsive disorder (OCD)
2. Specific phobia: If Jack's fear were limited to a specific type of "dirty" object, this could be considered.
3. Generalized anxiety disorder: Though less likely than the above options, the element of constant worry could point towards this diagnosis.
4. Other anxiety disorders: Depending on the specific anxieties and compulsions involved, other anxiety disorders might be considered, but more information would be needed for a definitive assessment.

Please note: This analysis is based solely on the limited information provided in the vignette. A comprehensive evaluation by a mental health professional is crucial for accurate diagnosis and appropriate treatment.

2015 version for PCPs and Psychology Doctoral trainees

Vignette 1 (2015)

Unless otherwise stated, Jack's symptoms below are not because of another psychology condition. They are not due to a general medical condition. They are not due to substance use. The symptoms, unless stated, have been present for more than five years and cause him significant distress.

Jack, a middle-aged man, thought about pushing the lady next to him onto the subway tracks. He was afraid by the thought and the fear that he may act on it, so he immediately left the subway and decided to walk home. Jack remained worried and continued to visually replay the situation to ensure that he did not actually harm the lady. Jack frequently finds himself worrying that he may want to or will harm others and these thoughts greatly upset him. He knows that his thoughts come from within his own mind and are excessive in nature. However, even knowing this he remains upset by the thoughts and is not able to stop them.

Based on the vignette which option most likely applies? If more than one, please mark all options that may apply. If you marked more than one of the above, please rank your choices in order of likelihood.
Lastly, use behavioral signs and symptoms from the vignette to step by step deduce the correct response and show me your reasoning for your choice of most likely option.

1. Adjustment disorder
2. Agoraphobia with panic disorder
3. Agoraphobia without panic disorder
4. Alcohol abuse
5. Alcohol dependence
6. Anger management issues

7. Anorexia nervosa
8. Antisocial personality disorder
9. Asperger's disorder
10. Attention deficit hyperactivity disorder
11. Autism disorder
12. Avoidant personality disorder
13. Bipolar I disorder
14. Bipolar II disorder
15. Body dysmorphic disorder
16. Borderline personality disorder
17. Bulimia Nervosa
18. Delusional disorder
19. Due to a general medical condition
20. Generalized anxiety disorder
21. Histrionic personality disorder
22. Hypochondriasis
23. Impulse control disorder, NOS
24. Intermittent explosive disorder
25. Major depression disorder
26. Marital problems
27. Narcissistic personality disorder
28. No disorder/condition
29. Obsessive-compulsive disorder
30. Obsessive-compulsive personality disorder
31. Panic disorder
32. Paranoid personality disorder
33. Pedophilia
34. Posttraumatic stress disorder
35. Primary Insomnia
36. Psychosis
37. Sexual identity confusion
38. Schizophrenia
39. Schizoid personality disorder
40. Schizotypal personality disorder
41. Social phobia/Social anxiety disorder
42. Somatization disorder
43. Specific phobia
44. Strong religious values
45. Tourette's
46. Trichotillomania
47. Other (specify)

LLM responses to vignette 1 (2015).

V1 (2015). ChatGPT-4

Jack's symptoms, as described in the vignette, align most closely with Obsessive-Compulsive Disorder (OCD). Here is the reasoning:

1. **Intrusive thoughts:** Jack experiences unwanted thoughts about pushing a lady onto the subway tracks.
2. **Anxiety due to these thoughts:** The thoughts are causing him significant distress and fear.
3. **Recognition that these thoughts are a product of his own mind:** Jack is aware that the thoughts are his own and not imposed from outside sources.
4. **Attempts to suppress or neutralize thoughts:** Jack tries to reassure himself by replaying the situation to ensure he did not act on the thoughts.
5. **Significant distress:** The symptoms cause Jack considerable distress.
6. **Duration:** The symptoms have been present for more than five years.

This assessment rules out other disorders because:

- There is no indication of adjustment issues (1), agoraphobia (2, 3), alcohol-related problems (4, 5), or specific problems with anger management (6).
- There's no evidence suggesting eating disorders like anorexia nervosa (7) or bulimia nervosa (17).
- Antisocial personality disorder (8) does not fit as Jack is concerned about his thoughts and the potential harm to others.
- Disorders on the autism spectrum (9, 11) do not typically present with intrusive harm thoughts as a primary symptom.
- ADHD (10) does not involve intrusive thoughts of harm.
- Avoidant personality disorder (12), bipolar disorders (13, 14), body dysmorphic disorder (15), and borderline personality disorder (16) are not characterized by intrusive thoughts of the type Jack has.
- The symptoms do not suggest delusional disorder (18) since Jack can recognize the irrational nature of his thoughts.
- Generalized anxiety disorder (20) typically involves a broader range of anxieties rather than specific intrusive thoughts.
- The vignette does not describe symptoms typical of the other disorders listed (19, 21-47).

Based on the information given, OCD (29) appears to be the most likely diagnosis, followed by Obsessive-Compulsive Personality Disorder (OCPD, 30). However, OCPD usually involves a pattern of preoccupation with orderliness, perfectionism, and control, rather than intrusive thoughts about harm.

Thus, the most likely option based on the vignette is:

1. Obsessive-compulsive disorder (29)

While other diagnoses could be considered in a full clinical assessment, based on the information given, OCD is the most fitting diagnosis.

V1 (2015). Llama 3

Based on the vignette, I believe the most likely option is:

29. Obsessive-compulsive disorder

Here's my step-by-step reasoning:
1. Jack experiences intrusive thoughts about harming others, which are distressing to him. This is a classic symptom of obsessive-compulsive disorder (OCD).

2. Jack recognizes that these thoughts come from within his own mind and are excessive in nature, indicating insight into the irrationality of his thoughts.

3. Despite this insight, Jack remains upset by the thoughts and is unable to stop them, which is consistent with the ego-dystonic nature of OCD symptoms.

4. Jack's behavior of visually replaying the situation to ensure he didn't harm the lady is a common compulsion seen in OCD, aimed at reducing anxiety.

5. The presence of significant distress and impairment in daily life (e.g., leaving the subway and deciding to walk home) also supports an OCD diagnosis.

I ranked my choices as follows:
1. Obsessive-compulsive disorder (29)
2. Generalized anxiety disorder (20)
While Jack's symptoms do involve excessive worry and anxiety, the specific nature of his intrusive thoughts and compulsions points more strongly towards an OCD diagnosis.

V1 (2015). Gemini pro

Analyzing Jack's symptoms:

Based on the information provided, here's an analysis of Jack's symptoms and potential diagnoses:

Symptoms:
- Intrusive thoughts of harming others
- Fear of acting on these thoughts
- Anxiety and distress caused by the thoughts
- Knowing the thoughts are excessive and unwanted
- Inability to control the thoughts

Behavioral signs:
- Leaving the subway due to fear
- Repetitive mental replaying of the situation

Exclusions:
- Not caused by another psychological condition
- Not due to a general medical condition
- Not due to substance use
- Symptoms present for more than five years

- Significant distress caused by the symptoms

Potential diagnoses:
Based on the information, several diagnoses could potentially apply. Here are the most likely options, ranked in order of likelihood:

1. Obsessive-compulsive disorder (OCD): This is the most likely diagnosis given Jack's intrusive thoughts and anxiety. His fear of harming others is a common obsession in OCD, and the distress and inability to control the thoughts are also characteristic of the disorder.
2. Generalized anxiety disorder (GAD): While Jack's primary concern seems to be the intrusive thoughts, the information also mentions general worry and anxiety. This could indicate GAD, which often co-occurs with OCD.
3. Body dysmorphic disorder (BDD): Although less likely than the above options, BDD can sometimes manifest as intrusive thoughts about harming oneself or others, particularly if the individual has concerns about their own physical appearance. However, the information provided doesn't explicitly mention body image concerns, making this a less likely possibility.

Excluding other options:
- The other listed diagnoses are less likely due to the specific nature of Jack's symptoms and the information provided. For instance, diagnoses like depression, bipolar disorder, or personality disorders typically present with different symptom profiles. Additionally, the information excludes the possibility of substance-induced or medical causes.

Reasoning:
The presence of intrusive thoughts, fear, anxiety, and the inability to control these thoughts strongly points towards OCD as the primary diagnosis. The additional presence of general worry suggests the possibility of co-occurring GAD. While BDD can involve intrusive thoughts about harming others, the lack of information regarding body image concerns makes it a less likely possibility.

It is important to remember that this is just an analysis based on the limited information provided. A proper diagnosis can only be made by a qualified mental health professional after a thorough evaluation.

Vignette 2 (2015)

Unless otherwise stated, Jack’s symptoms below are not because of another psychology condition. They are not due to a general medical condition. They are not due to substance use. The symptoms, unless stated, have been present for more than five years and cause him significant distress.

Jack, a young adult, has been in a committed relationship with his girlfriend for over five years. He loves her very much and is attracted to her. Although he is not sexually attracted to men, Jack is preoccupied by thoughts that he may be gay and worries that he is not living an honest life. He knows that his thoughts come from within his own mind and are excessive in nature. However, even knowing this he remains upset by the thoughts and is not able to stop them.

Upon seeing men, Jack immediately assesses his body for any signs that he may be sexually aroused; when he finds no signs of arousal, he experiences momentary relief.

Based on the vignette which option most likely applies? If more than one, please mark all options that may apply. If you marked more than one of the above, please rank your choices in order of likelihood.
Lastly, use behavioral signs and symptoms from the vignette to step by step deduce the correct response and show me your reasoning for your choice of most likely option.

1. Adjustment disorder
2. Agoraphobia with panic disorder
3. Agoraphobia without panic disorder
4. Alcohol abuse
5. Alcohol dependence
6. Anger management issues
7. Anorexia nervosa
8. Antisocial personality disorder
9. Asperger's disorder
10. Attention deficit hyperactivity disorder
11. Autism disorder
12. Avoidant personality disorder
13. Bipolar I disorder
14. Bipolar II disorder
15. Body dysmorphic disorder
16. Borderline personality disorder
17. Bulimia Nervosa
18. Delusional disorder
19. Due to a general medical condition
20. Generalized anxiety disorder
21. Histrionic personality disorder
22. Hypochondriasis
23. Impulse control disorder, NOS
24. Intermittent explosive disorder
25. Major depression disorder
26. Marital problems
27. Narcissistic personality disorder
28. No disorder/condition
29. Obsessive-compulsive disorder
30. Obsessive-compulsive personality disorder
31. Panic disorder
32. Paranoid personality disorder
33. Pedophilia
34. Posttraumatic stress disorder
35. Primary Insomnia
36. Psychosis
37. Sexual identity confusion
38. Schizophrenia
39. Schizoid personality disorder
40. Schizotypal personality disorder
41. Social phobia/Social anxiety disorder

42. Somatization disorder
43. Specific phobia
44. Strong religious values
45. Tourette's
46. Trichotillomania
47. Other (specify)

LLM responses to vignette 2 (2015)

V2 (2015). ChatGPT-4

Based on the details provided in the vignette, Jack's symptoms are indicative of Obsessive-Compulsive Disorder (OCD). The key aspects that support this diagnosis are:

1. **Presence of Obsessions**: Jack is preoccupied by persistent thoughts about being gay, even though he does not have an attraction to men. This preoccupation can be considered an obsession, which is a hallmark of OCD.
2. **Awareness of the Excessive Nature of Thoughts**: Jack is aware that these thoughts come from within his mind and are excessive, suggesting insight into the irrational nature of his obsessions, consistent with OCD.
3. **Attempt to Neutralize with a Compulsion**: Jack checks for signs of arousal upon seeing men, which can be seen as a compulsion to neutralize his anxiety caused by the obsessive thoughts.
4. **Transient Relief from Anxiety**: The momentary relief he experiences when he finds no signs of arousal is also characteristic of the compulsive behavior in OCD.
5. **Significant Distress and Duration**: The symptoms have been present for more than five years and cause significant distress, fulfilling the criteria for the duration and impact on life that are necessary for an OCD diagnosis.

Based on these points, option 29 (Obsessive-compulsive disorder) is the most likely diagnosis. The behavioral signs and symptoms such as persistent, unwanted thoughts (obsessions) and behaviors that he feels driven to perform (compulsions) that are aimed at preventing or reducing anxiety or distress, not to provide pleasure or gratification, are typical of OCD.

Other conditions listed do not seem to fit as well because they lack the characteristic obsessive-compulsive features present in Jack's description. For example, an adjustment disorder would involve emotional or behavioral symptoms in response to an identifiable stressor, which doesn't match Jack's situation. Conditions like personality disorders, substance abuse, or mood disorders do not align with the specific obsessive and compulsive symptoms described.

Hence, option 29 (Obsessive-compulsive disorder) is the correct choice, and it is the single most likely diagnosis without a need for ranking against the other options provided.

V2 (2015). Llama 3

Based on the vignette, I believe the most likely option is:

29. Obsessive-compulsive disorder

Here's my step-by-step reasoning:
1. Jack is preoccupied with thoughts that he may be gay, despite knowing they come from his own mind and are excessive in nature. This suggests an intrusive thought pattern, which is a hallmark of obsessive-compulsive disorder (OCD).

2. Jack is upset by these thoughts and unable to stop them, indicating significant distress.

3. When seeing men, Jack immediately assesses his body for any signs of sexual arousal, which is a compulsive behavior aimed at reducing anxiety.

4. The momentary relief he experiences when he finds no signs of arousal suggests a temporary reduction in anxiety, which is often seen in OCD.

Ranking my choices in order of likelihood:1. 29. Obsessive-compulsive disorder2. 20. Generalized anxiety disorder (although the specific symptoms described are more consistent with OCD)I did not consider other options, such as adjustment disorder or marital problems, as the primary issue seems to be Jack's internal struggles with his thoughts and compulsive behaviors rather than an external stressor or relationship issue.

V2 (2015). Gemini pro

Choosing the Most Likely Diagnoses for Jack

Based on the information provided in the vignette, here's an analysis of potential diagnoses for Jack, ranked in order of likelihood:

1. Obsessive-Compulsive Disorder (OCD) with predominantly Obsessions:

- Strong Evidence: Jack experiences intrusive thoughts (obsessions) about his sexuality, causing him significant distress. He attempts to neutralize these thoughts with mental compulsions (checking for arousal) but ultimately feels unsatisfied and remains preoccupied. This aligns with the core features of OCD.
- Supporting Evidence: The long duration of symptoms (over five years) and the absence of other contributing factors (general medical condition, substance use) further strengthen the likelihood of OCD.

2. Sexual Identity Confusion/Questioning:

- Moderate Evidence: Jack expresses uncertainty about his sexual orientation, questioning whether he might be gay despite not experiencing sexual attraction towards men. This suggests potential exploration and confusion regarding his sexual identity.
- Supporting Evidence: His intrusive thoughts about being gay could be related to unresolved questions or anxieties about his sexuality. However, it's crucial to distinguish this from OCD-related obsessions, where the content is unwanted and distressing.

3. Body Dysmorphic Disorder (BDD):

- Moderate Evidence: Jack constantly assesses his body for signs of arousal after seeing men. This behavior could be interpreted as checking for perceived flaws related to his masculinity or sexual orientation, potentially indicating a BDD presentation focused on specific body areas.
- Weaker Evidence: The vignette doesn't explicitly mention body dissatisfaction or preoccupation with perceived physical flaws, which are core features of BDD. Further information is needed to solidify this diagnosis.

4. Generalized Anxiety Disorder (GAD):

- Moderate Evidence: Jack's distress about his intrusive thoughts and his inability to control them could also indicate generalized anxiety. He may worry excessively about various aspects of his life, not just his perceived sexual orientation.
- Weaker Evidence: The vignette focuses primarily on the specific content of Jack's intrusive thoughts related to sexuality. More information about his overall anxiety levels and worry patterns is needed for a clearer diagnosis.

Unlikely Diagnoses:

Several other options on the list can be ruled out based on the information provided. For instance, there's no evidence to support diagnoses like Agoraphobia, Bipolar disorder, Anorexia Nervosa, or Schizophrenia.

Conclusion:

Based on the available information, Obsessive-Compulsive Disorder (OCD) appears to be the most likely diagnosis for Jack, followed by Sexual Identity Confusion/Questioning, Body Dysmorphic Disorder, and Generalized Anxiety Disorder as potential contributing factors. A thorough clinical evaluation is necessary to confirm the diagnoses and determine the most appropriate course of treatment.

Vignette 3 (2015)

Unless otherwise stated, Jack's symptoms below are not because of another psychology condition. They are not due to a general medical condition. They are not due to substance use. The symptoms, unless stated, have been present for more than five years and cause him significant distress.

Jack, a middle-aged man, loved spending time with his nieces and nephews and was considered their "favorite uncle." However, he started having images of touching the children in a sexual manner. He had no desire to touch the children and did not experience any sexual arousal during the image, but the worry of "what if" remained. He knows that his thoughts come from within his own mind and are excessive in nature. However, even knowing this he remains upset by the thoughts and is not able to stop them. He now tries to avoid being with the children and refuses to spend time alone with them.

Based on the vignette which option most likely applies? If more than one, please mark all options that may apply. If you marked more than one of the above, please rank your choices in order of likelihood.
Lastly, use behavioral signs and symptoms from the vignette to step by step deduce the correct response and show me your reasoning for your choice of most likely option.

1. Adjustment disorder
2. Agoraphobia with panic disorder
3. Agoraphobia without panic disorder
4. Alcohol abuse
5. Alcohol dependence
6. Anger management issues
7. Anorexia nervosa
8. Antisocial personality disorder
9. Asperger's disorder
10. Attention deficit hyperactivity disorder
11. Autism disorder
12. Avoidant personality disorder
13. Bipolar I disorder
14. Bipolar II disorder
15. Body dysmorphic disorder
16. Borderline personality disorder
17. Bulimia Nervosa
18. Delusional disorder
19. Due to a general medical condition
20. Generalized anxiety disorder
21. Histrionic personality disorder
22. Hypochondriasis
23. Impulse control disorder, NOS
24. Intermittent explosive disorder
25. Major depression disorder
26. Marital problems
27. Narcissistic personality disorder
28. No disorder/condition
29. Obsessive-compulsive disorder
30. Obsessive-compulsive personality disorder
31. Panic disorder
32. Paranoid personality disorder
33. Pedophilia
34. Posttraumatic stress disorder
35. Primary Insomnia
36. Psychosis
37. Sexual identity confusion
38. Schizophrenia
39. Schizoid personality disorder
40. Schizotypal personality disorder
41. Social phobia/Social anxiety disorder
42. Somatization disorder
43. Specific phobia
44. Strong religious values

45. Tourette's
46. Trichotillomania
47. Other (specify)

LLM responses to vignette 3 (2015)

V3 (2015). ChatGPT-4: N/A

V3 (2015). Llama 3

Based on the vignette, I believe the most likely option is:
29. Obsessive-compulsive disorder

Here's my step-by-step reasoning:
1. The vignette states that Jack has recurring images of touching his nieces and nephews in a sexual manner, which causes him significant distress. This suggests an intrusive thought pattern, which is a hallmark symptom of obsessive-compulsive disorder (OCD).

2. Jack reports no desire to act on these thoughts and experiences no sexual arousal during them. This rules out pedophilia (option 33) as a primary diagnosis.

3. The vignette emphasizes that Jack knows the thoughts come from within his own mind and are excessive in nature. This self-awareness and recognition of the irrationality of his thoughts are common features of OCD.

4. Despite knowing that his thoughts are unwanted and excessive, Jack is unable to stop them, which further supports the diagnosis of OCD.

5. Jack's avoidance of being with his nieces and nephews, especially alone, is a behavioral sign of OCD. He is trying to avoid situations that might trigger his intrusive thoughts or make him feel anxious about acting on them.

While it's possible that Jack may also experience anxiety or fear related to his intrusive thoughts (which could be indicative of agoraphobia or social phobia), the primary symptoms described in the vignette point more strongly towards OCD.

Ranking my choices in order of likelihood:
1. Obsessive-compulsive disorder (29)
2. Generalized anxiety disorder (20)
3. Social phobia/Social anxiety disorder (41)
Please note that a thorough diagnostic evaluation would require more information and a comprehensive assessment to confirm the diagnosis.

V3 (2015). Gemini pro: N/A

Vignette 4 (2015)

Unless otherwise stated, Jack's symptoms below are not because of another psychology condition. They are not due to a general medical condition. They are not due to substance use. The symptoms, unless stated, have been present for more than five years and cause him significant distress.

Jack, a middle-aged, highly religious man, believes that one should not have any negative thoughts about religion. He greatly worries when he notices himself having such negative religious thoughts (i.e. why does God have bad things happen to good people?). When these "bad" thoughts occur, as they frequently do, he becomes distressed and fears God will punish him. Jack then prays repeatedly to himself until he feels safe from harm, this can go on for hours. He knows that his thoughts come from within his own mind and are excessive in nature. However, even knowing this he remains upset by the thoughts and is not able to stop them.

Based on the vignette which option most likely applies? If more than one, please mark all options that may apply. If you marked more than one of the above, please rank your choices in order of likelihood.
Lastly, use behavioral signs and symptoms from the vignette to step by step deduce the correct response and show me your reasoning for your choice of most likely option.

1. Adjustment disorder
2. Agoraphobia with panic disorder
3. Agoraphobia without panic disorder
4. Alcohol abuse
5. Alcohol dependence
6. Anger management issues
7. Anorexia nervosa
8. Antisocial personality disorder
9. Asperger's disorder
10. Attention deficit hyperactivity disorder
11. Autism disorder
12. Avoidant personality disorder
13. Bipolar I disorder
14. Bipolar II disorder
15. Body dysmorphic disorder
16. Borderline personality disorder
17. Bulimia Nervosa
18. Delusional disorder
19. Due to a general medical condition
20. Generalized anxiety disorder
21. Histrionic personality disorder
22. Hypochondriasis
23. Impulse control disorder, NOS
24. Intermittent explosive disorder
25. Major depression disorder
26. Marital problems
27. Narcissistic personality disorder
28. No disorder/condition

29. Obsessive-compulsive disorder
30. Obsessive-compulsive personality disorder
31. Panic disorder
32. Paranoid personality disorder
33. Pedophilia
34. Posttraumatic stress disorder
35. Primary Insomnia
36. Psychosis
37. Sexual identity confusion
38. Schizophrenia
39. Schizoid personality disorder
40. Schizotypal personality disorder
41. Social phobia/Social anxiety disorder
42. Somatization disorder
43. Specific phobia
44. Strong religious values
45. Tourette's
46. Trichotillomania
47. Other (specify)

LLM responses to vignette 4 (2015)

V4 (2015). ChatGPT-4

Based on the details provided in the vignette, the option that most likely applies to Jack's condition is:

29. Obsessive-compulsive disorder (OCD)

To deduce this, let's look at the behavioral signs and symptoms mentioned and analyze them step by step:

1. Jack experiences intrusive negative religious thoughts which he recognizes as distressing and unwanted (i.e., "why does God have bad things happen to good people?").
2. These thoughts are frequent and cause him significant distress, which is a hallmark of the obsessions seen in OCD.
3. His response to these thoughts is to engage in compulsive behavior, specifically repeated prayer, to alleviate the distress and prevent perceived harm. This ritualistic behavior is a common compulsive action in OCD.
4. He continues these prayers for hours, which indicates that the compulsions are time-consuming and significantly interfere with his normal routine, another characteristic of OCD.
5. Jack is aware that his thoughts are excessive and come from his own mind, demonstrating insight into the irrational nature of his obsessions and compulsions, which is often the case in individuals with OCD.

6. Lastly, the symptoms have been present for more than five years and cause significant distress, which meets the duration and impairment criteria typically associated with OCD.

Given these points, OCD is the most fitting diagnosis for Jack's symptoms. The other options listed do not align as closely with the symptoms described. For example, adjustment disorder involves emotional or behavioral symptoms in response to an identifiable stressor, which doesn't match Jack's long-standing pattern of thoughts and behaviors. Agoraphobia and panic disorder are not indicated as there's no mention of fear or avoidance of places where escape might be difficult. Alcohol-related disorders are not suggested by the information given. And personality disorders listed do not correspond with the described obsessions and compulsions.

Therefore, the reasoning points towards a diagnosis of Obsessive-compulsive disorder.

V4 (2015). Llama 3

Based on the vignette, I believe the most likely option is:
29. Obsessive-compulsive disorder

Here's my step-by-step reasoning:
1. Jack experiences recurring, unwanted thoughts (negative religious thoughts) that cause him significant distress.

2. He attempts to suppress or neutralize these thoughts by praying repeatedly to himself until he feels safe from harm.

3. This behavior is excessive and time-consuming (can go on for hours).

4. Jack recognizes that his thoughts come from within his own mind and are excessive in nature, but he remains upset by them and is unable to stop them.

These symptoms match the diagnostic criteria for Obsessive-Compulsive Disorder (OCD):
* Recurring, unwanted thoughts (obsessions)
* Repetitive behaviors or mental acts (compulsions) aimed at reducing anxiety or preventing a perceived danger
* Recognizing that the obsessions and compulsions are excessive or unreasonable
* Significant distress or impairment in daily life due to the symptoms

Ranking my choices in order of likelihood:
1. Obsessive-compulsive disorder (29)
2. Generalized anxiety disorder (20) - although Jack's symptoms are more specific to obsessive thoughts and compulsive behaviors, generalized anxiety could be considered as a possible diagnosis.

I did not choose other options because:
* Adjustment disorder (1) is unlikely, as Jack's symptoms have been present for more than five years.

* Agoraphobia with or without panic disorder (2, 3) is not supported by the vignette, as there is no mention of fear or avoidance of specific situations.
* Other options do not align with the specific symptoms described in the vignette.

V4 (2015). Gemini pro

Determining the Most Likely Diagnosis for Jack

Based on the information provided, Jack is most likely experiencing Obsessive-Compulsive Disorder (OCD). Here's a breakdown of the reasoning:

Behavioral Signs and Symptoms Supporting OCD:

- Obsessive thoughts: Jack experiences intrusive, unwanted thoughts about religion that he deems "bad" or "negative." This aligns with the obsessional component of OCD, where individuals experience persistent, unwanted thoughts, images, or urges.
- Compulsions: To alleviate anxiety caused by these thoughts, Jack engages in repetitive behaviors like praying for hours. This aligns with the compulsion aspect of OCD, where individuals feel driven to perform specific actions or rituals to reduce anxiety.
- Distress and functional impairment: The intrusive thoughts and compulsions cause Jack significant distress and interfere with his daily life. This aligns with the requirement for OCD diagnosis that the symptoms cause significant distress or impairment in functioning.
- Awareness of excessive thoughts: Jack recognizes that his thoughts are excessive and unreasonable, which is a common feature of OCD.

Why other options are less likely:

- Adjustment disorder: While Jack's distress may be related to religious beliefs, the duration exceeding five years makes this diagnosis less likely.
- Panic disorder/agoraphobia: These diagnoses focus on panic attacks and fear of specific situations, which are not prominent in Jack's presentation.
- Substance use disorders: The vignette provides no indication of substance use contributing to the symptoms.
- Other disorders: Many listed disorders do not align with the specific symptoms of intrusive thoughts, compulsions, and religious-themed obsessions.

Likelihood ranking:

1. Obsessive-compulsive disorder (OCD)
2. Generalized anxiety disorder (GAD): While possible, the presence of clear obsessions and compulsions makes OCD more likely.
3. Scrupulosity OCD: This subtype of OCD specifically focuses on religious obsessions and compulsions, further increasing its likelihood in Jack's case.

Therefore, the most likely diagnosis for Jack is Obsessive-Compulsive Disorder (OCD), potentially with a scrupulosity subtype. He would benefit from seeking professional help from a mental health professional specializing in OCD treatment.

Vignette 5 (2015)

Unless otherwise stated, Jack's symptoms below are not because of another psychology condition. They are not due to a general medical condition. They are not due to substance use. The symptoms, unless stated, have been present for more than five years and cause him significant distress.

Jack, a middle-aged man, constantly worries about dirt and germs. He knows that his thoughts come from within his own mind and are excessive in nature. However, even knowing this he remains upset by the thoughts and is not able to stop them. He is unable to complete many of his daily activities because he tries at all costs to avoid touching things he thinks may be dirty. However, if he does touch a "dirty" object, Jack will immediately wash his hands so that he will not catch a disease.

Based on the vignette which option most likely applies? If more than one, please mark all options that may apply. If you marked more than one of the above, please rank your choices in order of likelihood.
Lastly, use behavioral signs and symptoms from the vignette to step by step deduce the correct response and show me your reasoning for your choice of most likely option.

1. Adjustment disorder
2. Agoraphobia with panic disorder
3. Agoraphobia without panic disorder
4. Alcohol abuse
5. Alcohol dependence
6. Anger management issues
7. Anorexia nervosa
8. Antisocial personality disorder
9. Asperger's disorder
10. Attention deficit hyperactivity disorder
11. Autism disorder
12. Avoidant personality disorder
13. Bipolar I disorder
14. Bipolar II disorder
15. Body dysmorphic disorder
16. Borderline personality disorder
17. Bulimia Nervosa
18. Delusional disorder
19. Due to a general medical condition
20. Generalized anxiety disorder
21. Histrionic personality disorder
22. Hypochondriasis
23. Impulse control disorder, NOS
24. Intermittent explosive disorder
25. Major depression disorder
26. Marital problems
27. Narcissistic personality disorder
28. No disorder/condition
29. Obsessive-compulsive disorder
30. Obsessive-compulsive personality disorder
31. Panic disorder
32. Paranoid personality disorder

33. Pedophilia
34. Posttraumatic stress disorder
35. Primary Insomnia
36. Psychosis
37. Sexual identity confusion
38. Schizophrenia
39. Schizoid personality disorder
40. Schizotypal personality disorder
41. Social phobia/Social anxiety disorder
42. Somatization disorder
43. Specific phobia
44. Strong religious values
45. Tourette's
46. Trichotillomania
47. Other (specify)

LLM responses to vignette 5 (2015)

V5 (2015). ChatGPT-4

Based on the vignette provided, the behavioral signs and symptoms described for Jack point towards a diagnosis of Obsessive-Compulsive Disorder (OCD).

Let's analyze the information given:

- Jack has a constant worry about dirt and germs.
- He recognizes these thoughts as his own and understands that they are excessive.
- These thoughts cause him significant distress and impair his ability to complete daily activities.
- He engages in compulsive hand washing after touching objects he perceives as "dirty" to prevent catching a disease.
- The symptoms have been present for more than five years.

The symptoms described do not align with Adjustment Disorder, which typically occurs in response to a significant life change or stressor and does not typically include the specific obsessive thoughts and compulsive behaviors described. Agoraphobia, with or without panic disorder, is characterized by a fear of places and situations that might cause panic, helplessness, or embarrassment, and does not specifically involve obsessions and compulsions related to cleanliness or germs.

Similarly, disorders related to substance use (Alcohol Abuse and Alcohol Dependence), personality disorders, mood disorders (such as Bipolar Disorder and Major Depression), eating disorders (Anorexia Nervosa, Bulimia Nervosa), and psychotic disorders (such as Schizophrenia) are not consistent with the behaviors Jack is exhibiting.

OCD, on the other hand, is characterized by the presence of obsessions, which are persistent, unwanted thoughts, and compulsions, which are repetitive behaviors that an individual feels

driven to perform in response to an obsession. Jack's awareness of his thoughts being excessive and his repeated hand washing after coming into contact with perceived contaminants are hallmark symptoms of OCD.

Thus, the most likely diagnosis, based on the information provided, would be:

29. Obsessive-compulsive disorder (OCD)

Jack's symptoms align with the DSM-5 criteria for OCD, which includes the presence of obsessions, compulsions, or both, that are time-consuming or cause clinically significant distress or impairment in social, occupational, or other important areas of functioning.

The ranking would be:

1. Obsessive-compulsive disorder

There are no other conditions that closely align with the symptoms described as well as OCD does, so no second choice is necessary.

V5 (2015). Llama 3

Based on the vignette, I believe the most likely option is:
29. Obsessive-compulsive disorder

Here's my step-by-step reasoning:
1. The vignette states that Jack constantly worries about dirt and germs, which suggests an excessive preoccupation with cleanliness.

2. Jack knows that his thoughts are excessive and come from within his own mind, but he's unable to stop them. This indicates a lack of control over his thoughts, which is a key feature of obsessive-compulsive disorder (OCD).

3. Jack's avoidance of touching things he thinks may be dirty and his immediate hand-washing after touching a "dirty" object suggest compulsive behaviors aimed at reducing anxiety.

4. The vignette mentions that Jack is unable to complete many of his daily activities due to his avoidance behaviors, which implies significant distress and impairment in his daily life.

Other options that might be considered, but are less likely, are:
* 20. Generalized anxiety disorder: While Jack does experience excessive worry, the specificity of his concerns (dirt and germs) and the presence of compulsive behaviors (hand-washing) make OCD a more fitting diagnosis.
* 22. Hypochondriasis: Although Jack fears catching diseases, his primary concern is dirt and germs, rather than having a specific illness or physical symptoms.

I ranked these options in order of likelihood as follows:
1. Obsessive-compulsive disorder
2. Generalized anxiety disorder

3. Hypochondriasis

V5 (2015). Gemini pro

Analysis of Jack's Symptoms:

Based on the information provided, Jack's symptoms are most consistent with Obsessive-Compulsive Disorder (OCD). Here's a breakdown of the reasoning:

Symptoms indicative of OCD:

- Obsessions: Jack constantly worries about dirt and germs, even though he knows these thoughts are excessive. This indicates the presence of intrusive and unwanted thoughts, a hallmark of obsessions in OCD.
- Compulsions: Jack engages in handwashing to alleviate his anxiety about germs. This repetitive behavior aimed at reducing anxiety is a clear sign of compulsions in OCD.
- Significant distress and impairment: Jack's symptoms cause him significant distress and interfere with his daily activities, preventing him from completing many tasks. This indicates the severity and impact of his OCD.

Additional factors supporting the diagnosis of OCD:

- Duration of symptoms: The symptoms have been present for more than five years, meeting the duration criteria for OCD diagnosis.
- Non-psychotic nature: There is no mention of hallucinations or delusions, suggesting the absence of psychosis.
- No substance use: The symptoms are not attributed to substance use.
- No other medical conditions: The symptoms are not due to another medical condition.

Other diagnoses considered:

- Generalized Anxiety Disorder (GAD): While Jack experiences anxiety, the specific focus on contamination and the presence of compulsions point more towards OCD.
- Specific Phobia: Specific phobias involve intense fear of specific objects or situations, unlike OCD's broader focus on intrusive thoughts and compulsive behaviors.
- Hypochondriasis: This involves excessive preoccupation with having a serious illness, which is not the central theme in Jack's case. His primary concern is contamination and germs, not the fear of having a specific disease.

Therefore, based on the presented information, the most likely diagnosis for Jack is Obsessive-Compulsive Disorder (OCD).

Ranking of other potential diagnoses:

1. Generalized Anxiety Disorder (GAD): Less likely than OCD due to the specific focus on contamination and compulsions.
2. Specific Phobia: Less likely than OCD due to the broader focus of OCD symptoms.
3. Hypochondriasis: Less likely than OCD due to the different focus on contamination and germs rather than fear of specific illnesses.

Vignette 6 (2015)

Unless otherwise stated, Jack's symptoms below are not because of another psychology condition. They are not due to a general medical condition. They are not due to substance use. The symptoms, unless stated, have been present for more than five years and cause him significant distress.

Jack, a middle-aged man, worries that he says improper things (i.e. sexual comments or swears) when speaking with others. He knows that his thoughts come from within his own mind and are excessive in nature. However, even knowing this he remains upset by the thoughts and is not able to stop them. He constantly asks his co-workers and his wife when he is not at work if he has made any off-putting comments. Jack experiences temporary relief when they inform him he has made no such comments. Jack also often replays his conversations, in his head, many times to make sure that he did not say anything "bad."

Based on the vignette which option most likely applies? If more than one, please mark all options that may apply. If you marked more than one of the above, please rank your choices in order of likelihood.
Lastly, use behavioral signs and symptoms from the vignette to step by step deduce the correct response and show me your reasoning for your choice of most likely option.

1. Adjustment disorder
2. Agoraphobia with panic disorder
3. Agoraphobia without panic disorder
4. Alcohol abuse
5. Alcohol dependence
6. Anger management issues
7. Anorexia nervosa
8. Antisocial personality disorder
9. Asperger's disorder
10. Attention deficit hyperactivity disorder
11. Autism disorder
12. Avoidant personality disorder
13. Bipolar I disorder
14. Bipolar II disorder
15. Body dysmorphic disorder
16. Borderline personality disorder
17. Bulimia Nervosa
18. Delusional disorder
19. Due to a general medical condition
20. Generalized anxiety disorder
21. Histrionic personality disorder
22. Hypochondriasis
23. Impulse control disorder, NOS
24. Intermittent explosive disorder
25. Major depression disorder
26. Marital problems
27. Narcissistic personality disorder
28. No disorder/condition
29. Obsessive-compulsive disorder
30. Obsessive-compulsive personality disorder
31. Panic disorder

32. Paranoid personality disorder
33. Pedophilia
34. Posttraumatic stress disorder
35. Primary Insomnia
36. Psychosis
37. Sexual identity confusion
38. Schizophrenia
39. Schizoid personality disorder
40. Schizotypal personality disorder
41. Social phobia/Social anxiety disorder
42. Somatization disorder
43. Specific phobia
44. Strong religious values
45. Tourette's
46. Trichotillomania
47. Other (specify)

LLM responses to vignette 6 (2015)

V6 (2015). ChatGPT-4

Based on the information provided in the vignette, Jack's symptoms are consistent with a few possible disorders, but one stands out as the most likely:

1. **Obsessive-Compulsive Disorder (OCD) (Option 29)**: Jack has intrusive thoughts (in this case, of saying improper things), which he recognizes as his own and as excessive. Despite this recognition, he's unable to stop them and engages in repeated behaviors (asking for reassurance, replaying conversations in his head) that temporarily relieve his distress. These are classic symptoms of OCD, which is characterized by persistent thoughts (obsessions) and behaviors (compulsions) that the individual feels driven to perform.

The diagnostic reasoning for OCD based on the vignette includes:

- **Presence of Obsessions**: Jack worries excessively about saying improper things, which is an intrusive and persistent thought.
- **Presence of Compulsions**: He seeks reassurance from others and replays conversations, which are actions aimed at reducing the anxiety caused by the obsessions.
- **Recognition of Excessiveness**: Jack is aware that his thoughts are excessive.
- **Distress**: The symptoms cause significant distress in his life.
- **Duration**: The symptoms have been present for more than five years, fitting the chronic nature of OCD.

No other disorder listed in the options provided aligns as closely with the symptoms described in the vignette. For instance, adjustment disorder involves emotional and behavioral symptoms in

response to a identifiable stressor, and agoraphobia typically involves fear of places or situations from which escape might be difficult. While Jack's symptoms may cause distress in social situations, there is no indication that he fears the situations themselves or avoids them, which rules out social phobia/social anxiety disorder.

Therefore, Obsessive-Compulsive Disorder (Option 29) is the most likely diagnosis based on the information given.

V6 (2015). Llama 3

🦙

Based on the vignette, I believe the most likely option is:
29. Obsessive-compulsive disorder (OCD)

Here's my step-by-step reasoning:
1. Jack experiences excessive and intrusive thoughts (sexual comments or swears) that he knows come from his own mind, which is a hallmark symptom of OCD.

2. He is unable to stop these thoughts, despite knowing they are excessive, which suggests a lack of control over his thoughts, another key feature of OCD.

3. Jack seeks reassurance from others (co-workers and wife) about whether he has made off-putting comments, which is a common behavior in OCD individuals who seek constant reassurance to alleviate their anxiety.

4. He experiences temporary relief when others inform him he hasn't made any inappropriate comments, which suggests that his anxiety is temporarily reduced by the reassurance.

5. Jack replays conversations in his head many times to ensure he didn't say anything "bad," which is a classic example of rumination, a common symptom of OCD.

Ranking my choices in order of likelihood:
1. 29. Obsessive-compulsive disorder (OCD)
2. 41. Social phobia/Social anxiety disorder (although this could be a secondary diagnosis, as Jack's symptoms seem more focused on obsessive thoughts and behaviors rather than social anxiety per se)
3. 20. Generalized anxiety disorder (while Jack does exhibit anxiety, his symptoms are more specific to obsessive thoughts and behaviors, making OCD a more fitting diagnosis)
I did not choose other options because:
* There is no mention of panic attacks or agoraphobic avoidance, ruling out agoraphobia with or without panic disorder.
* There is no evidence of substance abuse or dependence.
* The symptoms do not seem to be related to a general medical condition or another psychological condition.
* The other options do not fit the specific pattern of symptoms described in the vignette.

V6 (2015). Gemini pro

Analysis of Jack's Symptoms:

Based on the information provided, Jack's symptoms are most consistent with Obsessive-Compulsive Disorder (OCD). Here's a breakdown of the reasoning:

Evidence for OCD:

- Obsessive thoughts: Jack experiences intrusive thoughts of a sexual or inappropriate nature that cause him significant distress.
- Compulsions: He engages in mental compulsions, such as replaying conversations and seeking reassurance from others, to try to alleviate his anxiety about saying something offensive.
- Chronic nature: The symptoms have been present for more than five years, which is characteristic of OCD.

Other possible diagnoses considered and ruled out:

- Generalized Anxiety Disorder (GAD): While Jack experiences anxiety, the specific content of his worries and the presence of compulsions point more towards OCD.
- Social Anxiety Disorder (SAD): Jack's anxiety is specifically related to the content of his thoughts and not a general fear of social situations.
- Body Dysmorphic Disorder (BDD): Jack's concerns are not focused on his physical appearance.
- Tourette's Syndrome: There is no evidence of involuntary tics, which are a key feature of Tourette's.

Likelihood ranking:

1. Obsessive-Compulsive Disorder (OCD)
2. Generalized Anxiety Disorder (GAD)
3. Social Anxiety Disorder (SAD)
4. Other diagnoses are significantly less likely.

Behavioral signs and symptoms supporting the diagnosis:

- Intrusive thoughts of a sexual or inappropriate nature.
- Mental compulsions (replaying conversations, seeking reassurance).
- Significant distress and impairment caused by the symptoms.
- Chronic nature of the symptoms (present for more than 5 years).

Additional notes:

- It's important to note that a definitive diagnosis can only be made by a qualified mental health professional through a comprehensive evaluation.
- The information provided only offers a preliminary assessment based on the limited information available.

Conclusion:

Based on the analysis, Obsessive-Compulsive Disorder (OCD) appears to be the most likely diagnosis for Jack's symptoms. However, further evaluation by a mental health professional is recommended for a definitive diagnosis and appropriate treatment recommendations.

Vignette 7 (2015)

Unless otherwise stated, Jack's symptoms below are not because of another psychology condition. They are not due to a general medical condition. They are not due to substance use. The symptoms, unless stated, have been present for more than five years and cause him significant distress.

Jack, a middle-aged man, worries that he frequently offends others due to his "bad breath." Jack has been told countless times that his breath does not smell but he constantly finds himself fearing that maybe *this time* it does and often breathes into his hands to smell his breath to make sure it is okay. In addition, Jack frequently searches online to find foods that may improve or worsen his breath and further bases his diet on his findings. He knows that his thoughts come from within his own mind and are excessive in nature. However, even knowing this he remains upset by the thoughts and is not able to stop them.

Based on the vignette which option most likely applies? If more than one, please mark all options that may apply. If you marked more than one of the above, please rank your choices in order of likelihood.
Lastly, use behavioral signs and symptoms from the vignette to step by step deduce the correct response and show me your reasoning for your choice of most likely option.

1. Adjustment disorder
2. Agoraphobia with panic disorder
3. Agoraphobia without panic disorder
4. Alcohol abuse
5. Alcohol dependence
6. Anger management issues
7. Anorexia nervosa
8. Antisocial personality disorder
9. Asperger's disorder
10. Attention deficit hyperactivity disorder
11. Autism disorder
12. Avoidant personality disorder
13. Bipolar I disorder
14. Bipolar II disorder
15. Body dysmorphic disorder
16. Borderline personality disorder
17. Bulimia Nervosa
18. Delusional disorder
19. Due to a general medical condition
20. Generalized anxiety disorder
21. Histrionic personality disorder
22. Hypochondriasis
23. Impulse control disorder, NOS
24. Intermittent explosive disorder
25. Major depression disorder
26. Marital problems
27. Narcissistic personality disorder
28. No disorder/condition
29. Obsessive-compulsive disorder
30. Obsessive-compulsive personality disorder

31. Panic disorder
32. Paranoid personality disorder
33. Pedophilia
34. Posttraumatic stress disorder
35. Primary Insomnia
36. Psychosis
37. Sexual identity confusion
38. Schizophrenia
39. Schizoid personality disorder
40. Schizotypal personality disorder
41. Social phobia/Social anxiety disorder
42. Somatization disorder
43. Specific phobia
44. Strong religious values
45. Tourette's
46. Trichotillomania
47. Other (specify)

LLM responses to vignette 7 (2015)

V7 (2015). ChatGPT-4

Jack's experience, as described in the vignette, is characterized by persistent and excessive worries about his breath, despite being told it is not an issue, and a behavioral pattern of checking his breath and searching for foods that could affect it. His recognition of these thoughts as excessive and originating within his own mind, combined with his inability to dismiss these thoughts, leads to significant distress. These details are indicative of an obsessive-compulsive spectrum disorder.

Considering the options provided:

1. Adjustment disorder: Unlikely, as there is no mention of a specific stressor Jack is reacting to, and the symptoms have been present for more than five years.

2-3. Agoraphobia with/without panic disorder: Unlikely, as there is no mention of fear or avoidance of situations due to concerns about panic.

4-5. Alcohol abuse/dependence: Unlikely, there is no mention of alcohol use.

6. Anger management issues: Unlikely, there is no mention of anger or difficulty controlling anger.
7. Anorexia nervosa: Unlikely, while there is a focus on diet, it is not related to weight or body image.
8. Antisocial personality disorder: Unlikely, Jack's behaviors are not indicative of a disregard for others or social norms.

9. Asperger's disorder: Unlikely, there is no mention of difficulties with social interactions or restricted interests.
10. Attention deficit hyperactivity disorder: Unlikely, as the primary concern is obsessive thoughts, not attention or hyperactivity.
11. Autism disorder: Unlikely, the description does not include difficulties with communication or repetitive behaviors.
12. Avoidant personality disorder: Unlikely, Jack's concerns are specific to his breath and not indicative of a pervasive pattern of social inhibition, feelings of inadequacy, or hypersensitivity to negative evaluation.

13-14. Bipolar I/II disorder: Unlikely, as there is no mention of manic or hypomanic episodes.

15. Body dysmorphic disorder: While Jack is preoccupied with an aspect of his physical appearance (his breath), body dysmorphic disorder typically involves a preoccupation with a perceived physical defect, which doesn't align exactly with Jack's symptoms.
16. Borderline personality disorder: Unlikely, as there is no evidence of unstable relationships, self-image, or impulsivity.
17. Bulimia Nervosa: Unlikely, no mention of binge eating or compensatory behaviors.
18. Delusional disorder: Unlikely, as Jack is aware his thoughts are a product of his mind and not based on reality.
19. Due to a general medical condition: Excluded by the premise of the vignette.
20. Generalized anxiety disorder: Possible, as Jack experiences excessive anxiety and worry. However, the specificity of his concerns to his breath might suggest a different disorder.
21. Histrionic personality disorder: Unlikely, no mention of attention-seeking behaviors or exaggerated emotions.
22. Hypochondriasis: Unlikely, as his concerns are not about having a serious illness.
23. Impulse control disorder, NOS: Unlikely, there is no mention of failure to resist a temptation, urge, or impulse that is harmful.
24. Intermittent explosive disorder: Unlikely, no mention of aggressive outbursts.
25. Major depression disorder: Unlikely, no mention of depressive symptoms.
26. Marital problems: Unlikely, no information is provided about marital issues.
27. Narcissistic personality disorder: Unlikely, no mention of a need for admiration or lack of empathy.
28. No disorder/condition: Unlikely, as the symptoms cause significant distress.
29. Obsessive-compulsive disorder (OCD): Very likely, as Jack exhibits obsessions (persistent thoughts about bad breath) and compulsions (checking his breath, researching foods).
30. Obsessive-compulsive personality disorder: Unlikely, as the focus is on Jack's thoughts and behaviors around a specific fear, not general personality traits of perfectionism or control.
31. Panic disorder: Unlikely, no mention of panic attacks.
32. Paranoid personality disorder: Unlikely, no evidence of pervasive distrust and suspicion.

33-47. Other disorders listed: Unlikely, based on the information provided in the vignette.

Based on this information, the most likely diagnosis for Jack, ranked in order of likelihood, would be:

1. Obsessive-compulsive disorder (OCD) - His symptoms align with the DSM-5 criteria for OCD, which includes the presence of obsessions, compulsions, or both. Jack's concern about his breath would constitute an obsession, and his repetitive checking and research behaviors are compulsions. The symptoms are time-consuming, cause clinically significant distress, and are not attributable to the physiological effects of a substance or another medical condition.
2. Generalized anxiety disorder - As a secondary possibility, since Jack exhibits a more generalized pattern of worry that could potentially fall under GAD, but the specific, ritualistic nature of his behaviors is more characteristic of OCD.

V7 (2015). Llama 3

Based on the vignette, I believe the most likely option is:
29. Obsessive-compulsive disorder

Here's my step-by-step reasoning:
1. Jack has a preoccupation with his breath, constantly worrying that it might smell bad despite being told otherwise. This excessive concern is a hallmark of obsessive thinking.

2. He engages in repetitive behaviors to alleviate his anxiety, such as breathing into his hands to check for bad breath and researching online for foods that might affect his breath. These actions are typical of compulsions.

3. Jack recognizes that his thoughts are excessive and irrational, but he can't stop them. This insight into the absurdity of his thoughts is characteristic of obsessive-compulsive disorder (OCD).

4. The symptoms have been present for more than five years and cause significant distress, which meets the duration and distress criteria for OCD.

Other options that might be considered, but are less likely, are:
* 15. Body dysmorphic disorder: While Jack has a preoccupation with his appearance (bad breath), his concerns are not primarily focused on physical appearance, but rather on the perceived impact of his breath on others.
* 20. Generalized anxiety disorder: Jack's worries about his breath could be seen as a manifestation of generalized anxiety, but the presence of clear obsessive thoughts and compulsive behaviors points more strongly towards OCD.
* 41. Social phobia/Social anxiety disorder: Jack's fears of offending others due to his breath could be interpreted as social anxiety, but the specific focus on bad breath and the presence of compulsions make OCD a more likely diagnosis.

Ranking my choices in order of likelihood:
1. Obsessive-compulsive disorder (29)
2. Body dysmorphic disorder (15)
3. Generalized anxiety disorder (20)
Please note that a definitive diagnosis should only be made by a qualified mental health professional after a thorough evaluation and assessment.

V7 (2015). Gemini pro

Applying the vignette to the DSM-5 criteria

Based on the information provided in the vignette, here's an analysis to determine the most likely diagnosis for Jack:

1. Body Dysmorphic Disorder (BDD):

- Symptoms: Jack's excessive preoccupation with his "bad breath," despite reassurance and lack of objective evidence, aligns with the core feature of BDD. His rituals (checking his breath, researching foods) and distress caused by these thoughts further support this diagnosis.
- Other options:
    - Obsessive-compulsive disorder (OCD): While repetitive thoughts and behaviors are present, the content of Jack's obsessions is focused on a perceived physical flaw, aligning more with BDD.
    - Hypochondriasis: This focuses on fear of having a serious illness, which doesn't seem to be Jack's primary concern.
    - Social anxiety disorder: While fear of social judgment is present, the specific focus on breath and the rituals point more towards BDD.

2. Social Anxiety Disorder (SAD):

- Symptoms: Jack's fear of offending others due to his breath and his resulting anxiety in social situations are key features of SAD.
- Other options:
    - Avoidant personality disorder: While Jack experiences some social anxiety, his primary concern is his breath, not a general fear of social situations.
    - Generalized anxiety disorder (GAD): Jack's anxiety seems specifically tied to social situations and his perceived flaw, suggesting SAD.

Therefore, the most likely diagnoses for Jack are:

1. Body Dysmorphic Disorder (BDD)
2. Social Anxiety Disorder (SAD)

BDD appears to be the more prominent diagnosis due to the intense focus on the perceived physical flaw and the extensive associated rituals and distress.

It's important to note that this analysis is based on limited information and a definitive diagnosis should be made by a qualified mental health professional through a comprehensive evaluation.

Vignette 8 (2015)

Unless otherwise stated, Jack's symptoms below are not because of another psychology condition. They are not due to a general medical condition. They are not due to substance use. The symptoms, unless stated, have been present for more than five years and cause him significant distress.

Jack, a middle-aged man, worries when things are not orderly and systematic. He becomes anxious when individuals move his belongings and feels he must immediately return the objects to their proper place. He also rearranges things that are not in order to place them how they

“should be.” When things are not in proper order Jack is unable to focus until the objects are back in their correct place. He knows that his thoughts come from within his own mind and are excessive in nature. However, even knowing this he remains upset by the thoughts and is not able to stop them.

Based on the vignette which option most likely applies? If more than one, please mark all options that may apply. If you marked more than one of the above, please rank your choices in order of likelihood.
Lastly, use behavioral signs and symptoms from the vignette to step by step deduce the correct response and show me your reasoning for your choice of most likely option.

1. Adjustment disorder
2. Agoraphobia with panic disorder
3. Agoraphobia without panic disorder
4. Alcohol abuse
5. Alcohol dependence
6. Anger management issues
7. Anorexia nervosa
8. Antisocial personality disorder
9. Asperger’s disorder
10. Attention deficit hyperactivity disorder
11. Autism disorder
12. Avoidant personality disorder
13. Bipolar I disorder
14. Bipolar II disorder
15. Body dysmorphic disorder
16. Borderline personality disorder
17. Bulimia Nervosa
18. Delusional disorder
19. Due to a general medical condition
20. Generalized anxiety disorder
21. Histrionic personality disorder
22. Hypochondriasis
23. Impulse control disorder, NOS
24. Intermittent explosive disorder
25. Major depression disorder
26. Marital problems
27. Narcissistic personality disorder
28. No disorder/condition
29. Obsessive-compulsive disorder
30. Obsessive-compulsive personality disorder
31. Panic disorder
32. Paranoid personality disorder
33. Pedophilia
34. Posttraumatic stress disorder
35. Primary Insomnia
36. Psychosis
37. Sexual identity confusion
38. Schizophrenia
39. Schizoid personality disorder

40. Schizotypal personality disorder
41. Social phobia/Social anxiety disorder
42. Somatization disorder
43. Specific phobia
44. Strong religious values
45. Tourette's
46. Trichotillomania
47. Other (specify)

LLM responses to vignette 8 (2015)

V8 (2015). ChatGPT-4

The vignette presents Jack, a middle-aged man, with a specific set of behavioral signs and symptoms. To deduce the most likely psychological condition, we'll analyze the key points provided:

1. **Significant Distress:** The symptoms cause him significant distress.
2. **Duration:** The symptoms have been present for more than five years.
3. **Desire for Order:** He is worried when things are not orderly and systematic.
4. **Anxiety from Displacement:** Becomes anxious when objects are moved and feels compelled to return them to their "proper" place.
5. **Reordering:** He rearranges disordered things to how they "should be."
6. **Focus Disruption:** Cannot focus until objects are back in their correct place.
7. **Awareness of Excess:** He is aware that his thoughts are from within and are excessive.
8. **Inability to Stop Thoughts:** Despite this awareness, he can't stop these thoughts.

Now, we will step by step eliminate conditions that do not match these symptoms and highlight those that do:

- **Generalized Anxiety Disorder (20)** usually involves excessive worry about various aspects of life, not specifically about order and placement of objects. While anxiety is present, the specific compulsions and distress related to object placement make this less likely.
- **Obsessive-Compulsive Disorder (29)** is characterized by both obsessions (intrusive, persistent thoughts) and compulsions (repetitive behaviors or mental acts that a person feels driven to perform in response to an obsession). Jack's distress when objects are not in place, his compulsion to reorder them, and his recognition of his thoughts as excessive and from his own mind align with OCD.
- **Obsessive-Compulsive Personality Disorder (30)** is a condition characterized by a chronic preoccupation with rules, orderliness, and control. While Jack exhibits a desire for order, OCPD does not typically include the compulsive behaviors (rearranging objects, inability to focus until order is restored) seen in OCD.
- **Other disorders listed (1-18, 21-28, 31-38, 40-47)** do not specifically align with Jack's described behaviors and thoughts related to orderliness, placement of objects, and associated distress and anxiety.

Based on the vignette, **Obsessive-Compulsive Disorder (29)** is the most likely diagnosis, considering the compulsive reordering of objects, the significant anxiety caused by disorder, the recognition of his own excessive thoughts, and the inability to dismiss or stop these thoughts even with that awareness. Obsessive-Compulsive Personality Disorder (30) could be a secondary consideration due to Jack's preoccupation with orderliness, but the presence of significant distress and compulsive behaviors aligns more closely with OCD.

Thus, the ranking of likelihood is as follows:

1. Obsessive-Compulsive Disorder (29)
2. Obsessive-Compulsive Personality Disorder (30)

The primary reason for selecting OCD over OCPD is the presence of compulsive behaviors and significant distress directly related to those compulsions, which are hallmarks of OCD.

V8 (2015). Llama 3

Based on the vignette, the most likely option that applies to Jack is:
29. Obsessive-compulsive disorder

Here's my step-by-step deduction and reasoning:
1. **Preoccupation with order and symmetry**: Jack worries when things are not orderly and systematic, which indicates a preoccupation with order and symmetry, a common theme in obsessive-compulsive disorder (OCD).

2. **Anxiety triggered by disorganization**: Jack becomes anxious when individuals move his belongings, which suggests that he experiences anxiety when his environment is not organized according to his standards.

3. **Compulsive behavior**: Jack feels compelled to immediately return objects to their proper place, indicating a strong urge to perform a specific behavior (rearranging) to reduce his anxiety.

4. **Inability to focus until objects are in order**: Jack is unable to focus until the objects are back in their correct place, which suggests that his anxiety and compulsions interfere with his daily functioning.

5. **Recognition of excessive thoughts**: Jack acknowledges that his thoughts are excessive in nature, which is consistent with the insight often seen in individuals with OCD.

6. **Lack of control over thoughts**: Despite recognizing the excessiveness of his thoughts, Jack remains upset by them and is unable to stop them, which is another characteristic of OCD.

Ranking my choices in order of likelihood:
1. 29. Obsessive-compulsive disorder
2. 30. Obsessive-compulsive personality disorder (although less likely, as Jack's symptoms seem more pronounced and interfering than those typically seen in OCPD)

I did not consider other options because they do not align with the symptoms described in the vignette. For example, there is no mention of panic attacks (2), substance abuse (4, 5), eating disorders (7, 17), or other conditions that might explain Jack's symptoms.

V8 (2015). Gemini pro

Analyzing Jack's symptoms:

Based on the information provided, Jack's symptoms point most likely towards Obsessive-compulsive disorder (OCD). Here's a step-by-step breakdown explaining the reasoning:

1. Preoccupation with order and system: Jack's excessive concern with orderliness and systematic arrangement of objects is a key characteristic of OCD. He experiences anxiety when things are not in their "proper" place and feels compelled to rearrange them. This preoccupation significantly impacts his daily life and causes him distress.

2. Repetitive behaviors: Jack engages in repetitive behaviors like returning objects to their designated spots and rearranging things to achieve order. These behaviors are aimed at reducing his anxiety but become a source of distress themselves due to their time-consuming nature.

3. Intrusive thoughts: Jack recognizes that his thoughts about order and arrangement are excessive and originate from within him. This awareness of the intrusive nature of his thoughts is consistent with OCD.

4. Distress and impairment: Jack's symptoms cause him significant distress and interfere with his daily functioning. He is unable to focus on other tasks until the objects are back in their "correct" place. This indicates the significant impact of his symptoms on his life.

5. Duration and absence of other causes: The information provided states that Jack's symptoms have been present for over five years, further supporting an OCD diagnosis. Additionally, the information specifies that the symptoms are not due to other psychological conditions, substance use, or general medical conditions, making other diagnoses less likely.
Therefore, based on the provided information and Jack's symptoms, the most likely diagnosis is Obsessive-compulsive disorder (OCD).

Other possible diagnoses:
While OCD is the most likely diagnosis, it's important to consider other possibilities:

- Obsessive-compulsive personality disorder (OCPD): This shares similarities with OCD but focuses on a pervasive need for orderliness, perfectionism, and control. However, the lack of intrusive thoughts and compulsive behaviors in the vignette makes OCPD less likely than OCD.
- Generalized anxiety disorder (GAD): GAD involves excessive worry and anxiety, but it typically doesn't manifest in specific rituals or compulsions like those seen in Jack's case.

- Autism spectrum disorder (ASD): Some individuals with ASD may exhibit strong preferences for order and routine, but the specific focus on intrusive thoughts and compulsive behaviors related to order points more towards OCD in Jack's case.

Further evaluation:
A comprehensive evaluation by a mental health professional is crucial for a definitive diagnosis. This would involve a thorough clinical interview, assessment of symptoms, and ruling out other potential causes.

2022 version for mental health providers in Guam

Vignette 1 (2022)

Unless otherwise stated, Jack's symptoms below are not because of another psychology condition. They are not due to a general medical condition. They are not due to substance use. The symptoms, unless stated, have been present for more than five years and cause him significant distress.

Jack, a middle-aged man, thought about pushing the lady next to him onto the subway tracks. He was afraid by the thought and the fear that he may act on it, so he immediately left the subway and decided to walk home. Jack remained worried and continued to visually replay the situation to ensure that he did not actually harm the lady. Jack frequently finds himself worrying that he may want to or will harm others and these thoughts greatly upset him. He knows that his thoughts come from within his own mind and are excessive in nature. However, even knowing this he remains upset by the thoughts and is not able to stop them.

Based on the vignette which option most likely applies? If more than one, please mark all options that may apply. If you marked more than one of the above, please rank your choices in order of likelihood.
Lastly, use behavioral signs and symptoms from the vignette to step by step deduce the correct response and show me your reasoning for your choice of most likely option.

1. Adjustment disorder
2. Agoraphobia with panic disorder
3. Agoraphobia without panic disorder
4. Alcohol abuse
5. Alcohol dependence
6. Anorexia nervosa
7. Antisocial personality disorder
8. Asperger's disorder
9. Attention deficit hyperactivity disorder
10. Autism disorder
11. Avoidant personality disorder
12. Bipolar I disorder
13. Bipolar II disorder
14. Body dysmorphic disorder
15. Borderline personality disorder

16. Bulimia Nervosa
17. Delusional disorder
18. Generalized anxiety disorder
19. Histrionic personality disorder
20. Hypochondriasis
21. Impulse control disorder, NOS
22. Major depression disorder
23. Marital problems
24. Narcissistic personality disorder
25. No disorder/condition
26. Obsessive-compulsive disorder
27. Obsessive-compulsive personality disorder
28. Panic disorder
29. Paranoid personality disorder
30. Pedophilia
31. Posttraumatic stress disorder
32. Primary Insomnia
33. Psychosis
34. Sexual identity confusion
35. Schizophrenia
36. Schizoid personality disorder
37. Schizotypal personality disorder
38. Social phobia/Social anxiety disorder
39. Somatization disorder
40. Specific phobia
41. Strong religious values
42. Tourette's
43. Trichotillomania
44. Other (specify)

LLM responses to vignette 1 (2022)

V1 (2022). ChatGPT-4

Based on the provided vignette, the most likely diagnosis for Jack is:

Obsessive-compulsive disorder (OCD) - Jack experiences intrusive thoughts about harming others, which cause him significant distress. He recognizes these thoughts as his own and excessive, indicating insight, yet he cannot control them. This aligns with OCD, which is characterized by obsessions (intrusive, distressing thoughts) and compulsions (behaviors aimed at reducing the anxiety associated with the obsessions), even though the compulsions are not clearly stated in the vignette.

The reasoning for this choice is as follows:

- The thoughts have been present for more than five years, which fits the chronic nature of OCD.
- The thoughts cause significant distress, a key feature of OCD.
- Jack has insight into the excessive nature of his thoughts, which is common in OCD.
- There is no mention of substance use or a general medical condition that could explain his symptoms.
- The thoughts are ego-dystonic (upsetting and not aligned with his desires), which is typical for OCD.
- There is no mention of behaviors that would indicate a disorder of personality, impulse control, eating, mood, or psychosis.

Other options like adjustment disorder, anxiety disorders (except OCD), mood disorders, personality disorders, eating disorders, and substance-related disorders do not fit the behavioral signs and symptoms described in the vignette as closely as OCD does.

Therefore, option 26 (Obsessive-compulsive disorder) is the most likely diagnosis. There is no mention of any other symptoms that would suggest a different or comorbid condition, hence no other options are marked.

V1 (2022). Llama 3

Based on the vignette, the most likely option is:
26. Obsessive-compulsive disorder (OCD)

Here's my step-by-step reasoning:
1. The vignette mentions that Jack has recurring thoughts about harming others, which are distressing to him and cause significant anxiety.

2. These thoughts are recognized by Jack as coming from his own mind and are excessive in nature, indicating an internal struggle with unwanted thoughts.

3. Despite knowing that these thoughts are not based in reality, Jack is unable to stop them and is greatly upset by them.

4. The vignette also highlights Jack's repetitive behavior of visually replaying the situation to ensure he didn't harm the lady, which suggests a compulsion to engage in mental checking.

These symptoms are consistent with the diagnostic criteria for Obsessive-Compulsive Disorder (OCD), which includes:
* Recurring, intrusive, and distressing thoughts (obsessions)
* Repetitive behaviors or mental acts (compulsions) aimed at reducing anxiety or preventing a perceived threat
* Recognition that the obsessions or compulsions are excessive or unreasonable
* Significant distress or impairment in daily life

Ranking my choices in order of likelihood:
1. 26. Obsessive-compulsive disorder (OCD)

2. 18. Generalized anxiety disorder (GAD) - While Jack's symptoms do involve anxiety, the specific features of OCD (intrusive thoughts, compulsions, and recognition of excessiveness) make OCD a more likely diagnosis.
3. 28. Panic disorder - Although Jack experiences fear and anxiety, there is no mention of panic attacks or specific phobias, making OCD a more fitting diagnosis.

No other options appear to match the symptoms described in the vignette.

V1 (2022). Gemini pro

Analysis of Jack's symptoms:

Based on the information provided, several options could potentially apply to Jack's situation. Here's a breakdown:

Most Likely:

- 26. Obsessive-compulsive disorder (OCD): Jack's intrusive thoughts about harming others and his subsequent anxiety and rumination are highly suggestive of OCD. His awareness that the thoughts are excessive and unwanted further supports this possibility.
- 38. Social phobia/Social anxiety disorder: Jack's fear of acting on his intrusive thoughts and the subsequent avoidance of the subway environment aligns with social anxiety symptoms. The fear of embarrassment or scrutiny associated with acting on the intrusive thoughts could be a driving factor in his anxiety.

Less Likely, but possible:

- 18. Generalized anxiety disorder (GAD): While Jack experiences significant anxiety, the specific content of his worries (harming others) and the presence of intrusive thoughts lean more towards OCD. However, GAD cannot be entirely ruled out.
- 21. Impulse control disorder, NOS: Jack's fear of acting on his impulses suggests a difficulty controlling them, which aligns with impulse control disorders. However, the specific nature of his intrusive thoughts points more towards OCD.

Unlikely:

- The remaining options are unlikely due to the absence of supporting symptoms. For instance, there is no indication of substance use, medical conditions, or other psychological conditions that could explain Jack's symptoms.

Ranking of options:

1. Obsessive-compulsive disorder (OCD)
2. Social phobia/Social anxiety disorder
3. Generalized anxiety disorder (GAD)
4. Impulse control disorder, NOS

Reasoning:

The presence of intrusive thoughts, anxiety about acting on those thoughts, and attempts to neutralize the anxiety through mental rituals (replaying the situation) are highly suggestive of OCD. Jack's fear of social scrutiny and the avoidance of the subway further support the possibility of social anxiety. While GAD presents with generalized anxiety, the specific content and characteristics of Jack's worries point more towards OCD. Finally, although the fear of acting on impulses suggests an impulse control issue, the nature of the intrusive thoughts aligns more closely with OCD.

Conclusion:

Based on the available information, OCD appears to be the most likely diagnosis for Jack. However, social anxiety and, to a lesser extent, GAD, remain possibilities. A comprehensive evaluation by a mental health professional is recommended for a definitive diagnosis and appropriate treatment plan.

Vignette 2 (2022)

Unless otherwise stated, Jack's symptoms below are not because of another psychology condition. They are not due to a general medical condition. They are not due to substance use. The symptoms, unless stated, have been present for more than five years and cause him significant distress.

Jack, a young adult, has been in a committed relationship with his girlfriend for over five years. He loves her very much and is attracted to her. Although he is not sexually attracted to men, Jack is preoccupied by thoughts that he may be gay and worries that he is not living an honest life. He knows that his thoughts come from within his own mind and are excessive in nature. However, even knowing this he remains upset by the thoughts and is not able to stop them. Upon seeing men, Jack immediately assesses his body for any signs that he may be sexually aroused; when he finds no signs of arousal, he experiences momentary relief.

Based on the vignette which option most likely applies? If more than one, please mark all options that may apply. If you marked more than one of the above, please rank your choices in order of likelihood.
Lastly, use behavioral signs and symptoms from the vignette to step by step deduce the correct response and show me your reasoning for your choice of most likely option.

1. Adjustment disorder
2. Agoraphobia with panic disorder
3. Agoraphobia without panic disorder
4. Alcohol abuse
5. Alcohol dependence
6. Anorexia nervosa
7. Antisocial personality disorder
8. Asperger's disorder
9. Attention deficit hyperactivity disorder
10. Autism disorder

11. Avoidant personality disorder
12. Bipolar I disorder
13. Bipolar II disorder
14. Body dysmorphic disorder
15. Borderline personality disorder
16. Bulimia Nervosa
17. Delusional disorder
18. Generalized anxiety disorder
19. Histrionic personality disorder
20. Hypochondriasis
21. Impulse control disorder, NOS
22. Major depression disorder
23. Marital problems
24. Narcissistic personality disorder
25. No disorder/condition
26. Obsessive-compulsive disorder
27. Obsessive-compulsive personality disorder
28. Panic disorder
29. Paranoid personality disorder
30. Pedophilia
31. Posttraumatic stress disorder
32. Primary Insomnia
33. Psychosis
34. Sexual identity confusion
35. Schizophrenia
36. Schizoid personality disorder
37. Schizotypal personality disorder
38. Social phobia/Social anxiety disorder
39. Somatization disorder
40. Specific phobia
41. Strong religious values
42. Tourette's
43. Trichotillomania
44. Other (specify)

LLM responses to vignette 2 (2022)

V2 (2022). ChatGPT-4

The description provided of Jack's experience indicates he is dealing with a persistent, distressing preoccupation with a concern about his sexual orientation, despite his long-term relationship with a woman whom he loves and finds attractive. This preoccupation involves excessive thoughts and self-monitoring for signs of arousal when he sees men, followed by relief when he does not find such signs. The key points here are the presence of intrusive thoughts, the understanding that these thoughts are excessive, and the significant distress and preoccupation they cause. There is no indication that he has a fear of places where escape might be difficult (agoraphobia), nor does he exhibit symptoms of a panic disorder. There's also

no indication of issues with alcohol, eating disorders, personality disorders, psychosis, mood disorders, impulse control issues, or other conditions listed.

Given this information, the most likely option from the list provided would be:

26. Obsessive-compulsive disorder (OCD)

OCD is characterized by the presence of obsessions, which are intrusive and unwanted thoughts that cause significant anxiety or distress, and compulsions, which are behaviors an individual feels compelled to perform in order to reduce anxiety or prevent some dreaded event or situation. In Jack's case, his obsession is the thought of being gay, and his compulsion is the checking for signs of arousal. This ritualistic checking provides temporary relief from his anxiety, which is consistent with the cycle of OCD. There are no other disorders on the list that are typically characterized by these particular types of obsessions and compulsions.

To deduce this, let's analyze step by step:

- **Long duration and significant distress:** The symptoms have been present for more than five years and cause significant distress, fitting the chronic nature of OCD.
- **Intrusive thoughts:** Jack’s preoccupation with the thought that he may be gay, despite evidence to the contrary, is akin to an intrusive thought.
- **Recognition of excessiveness:** Jack is aware that these thoughts are excessive and originate from within his mind, which aligns with the insight often seen in OCD.
- **Checking behaviors:** The act of checking his body for signs of arousal is a compulsion aimed at alleviating the distress caused by the obsession.
- **Temporary relief:** The relief Jack experiences after finding no signs of arousal is typical of the temporary respite compulsions can provide in OCD.

Based on the information provided, this is the most fitting diagnosis, and there is no indication that other disorders listed would be more appropriate to consider.

V2 (2022). Llama 3

Based on the vignette, the most likely option that applies is:
26. Obsessive-compulsive disorder (OCD)

Here's my step-by-step reasoning:
1. Jack is preoccupied with thoughts that he may be gay, despite knowing that these thoughts are excessive and come from within his own mind. This suggests an obsessive thought pattern.
2. He is upset by these thoughts and unable to stop them, which indicates significant distress.
3. Upon seeing men, Jack immediately assesses his body for any signs of sexual arousal, which suggests a compulsive behavior aimed at reducing anxiety or uncertainty.
4. When he finds no signs of arousal, he experiences momentary relief, which implies that the compulsive behavior is temporarily alleviating his anxiety.

These symptoms are consistent with OCD, which is characterized by recurring, intrusive thoughts (obsessions) and repetitive behaviors (compulsions) that an individual feels compelled to perform in response to these thoughts.

Ranking my choices in order of likelihood:
1. Obsessive-compulsive disorder (OCD)
2. Generalized anxiety disorder (18) - Although Jack's symptoms don't entirely fit the criteria for GAD, his excessive worrying and anxiety could be considered as a possible alternative diagnosis.

I did not consider other options, such as sexual identity confusion (34), because the vignette explicitly states that Jack is not sexually attracted to men and is committed to his girlfriend.

V2 (2022). Gemini pro

Analysis of Jack's symptoms:

Based on the information provided, several options could potentially apply to Jack's situation. Here's a breakdown:

Most Likely:

- 26. Obsessive-compulsive disorder (OCD): Jack's intrusive thoughts about harming others and his subsequent anxiety and rumination are highly suggestive of OCD. His awareness that the thoughts are excessive and unwanted further supports this possibility.
- 38. Social phobia/Social anxiety disorder: Jack's fear of acting on his intrusive thoughts and the subsequent avoidance of the subway environment aligns with social anxiety symptoms. The fear of embarrassment or scrutiny associated with acting on the intrusive thoughts could be a driving factor in his anxiety.

Less Likely, but possible:

- 18. Generalized anxiety disorder (GAD): While Jack experiences significant anxiety, the specific content of his worries (harming others) and the presence of intrusive thoughts lean more towards OCD. However, GAD cannot be entirely ruled out.
- 21. Impulse control disorder, NOS: Jack's fear of acting on his impulses suggests a difficulty controlling them, which aligns with impulse control disorders. However, the specific nature of his intrusive thoughts points more towards OCD.

Unlikely:

- The remaining options are unlikely due to the absence of supporting symptoms. For instance, there is no indication of substance use, medical conditions, or other psychological conditions that could explain Jack's symptoms.

Ranking of options:

1. Obsessive-compulsive disorder (OCD)
2. Social phobia/Social anxiety disorder
3. Generalized anxiety disorder (GAD)
4. Impulse control disorder, NOS

Reasoning:

The presence of intrusive thoughts, anxiety about acting on those thoughts, and attempts to neutralize the anxiety through mental rituals (replaying the situation) are highly suggestive of OCD. Jack's fear of social scrutiny and the avoidance of the subway further support the possibility of social anxiety. While GAD presents with generalized anxiety, the specific content and characteristics of Jack's worries point more towards OCD. Finally, although the fear of acting on impulses suggests an impulse control issue, the nature of the intrusive thoughts aligns more closely with OCD.

Conclusion:

Based on the available information, OCD appears to be the most likely diagnosis for Jack. However, social anxiety and, to a lesser extent, GAD, remain possibilities. A comprehensive evaluation by a mental health professional is recommended for a definitive diagnosis and appropriate treatment plan.

Vignette 3 (2022)

Unless otherwise stated, Jack's symptoms below are not because of another psychology condition. They are not due to a general medical condition. They are not due to substance use. The symptoms, unless stated, have been present for more than five years and cause him significant distress.

Jack, a middle-aged man, loved spending time with his nieces and nephews and was considered their "favorite uncle." However, he started having images of touching the children in a sexual manner. He had no desire to touch the children and did not experience any sexual arousal during the image, but the worry of "what if" remained. He knows that his thoughts come from within his own mind and are excessive in nature. However, even knowing this he remains upset by the thoughts and is not able to stop them. He now tries to avoid being with the children and refuses to spend time alone with them.

Based on the vignette which option most likely applies? If more than one, please mark all options that may apply. If you marked more than one of the above, please rank your choices in order of likelihood.
Lastly, use behavioral signs and symptoms from the vignette to step by step deduce the correct response and show me your reasoning for your choice of most likely option.

1. Adjustment disorder
2. Agoraphobia with panic disorder
3. Agoraphobia without panic disorder
4. Alcohol abuse
5. Alcohol dependence
6. Anorexia nervosa
7. Antisocial personality disorder
8. Asperger's disorder
9. Attention deficit hyperactivity disorder
10. Autism disorder

11. Avoidant personality disorder
12. Bipolar I disorder
13. Bipolar II disorder
14. Body dysmorphic disorder
15. Borderline personality disorder
16. Bulimia Nervosa
17. Delusional disorder
18. Generalized anxiety disorder
19. Histrionic personality disorder
20. Hypochondriasis
21. Impulse control disorder, NOS
22. Major depression disorder
23. Marital problems
24. Narcissistic personality disorder
25. No disorder/condition
26. Obsessive-compulsive disorder
27. Obsessive-compulsive personality disorder
28. Panic disorder
29. Paranoid personality disorder
30. Pedophilia
31. Posttraumatic stress disorder
32. Primary Insomnia
33. Psychosis
34. Sexual identity confusion
35. Schizophrenia
36. Schizoid personality disorder
37. Schizotypal personality disorder
38. Social phobia/Social anxiety disorder
39. Somatization disorder
40. Specific phobia
41. Strong religious values
42. Tourette's
43. Trichotillomania
44. Other (specify)

LLM responses to vignette 3 (2022)

V3 (2022). ChatGPT-4: N/A

V3 (2022). Llama 3

Based on the vignette, the most likely option that applies to Jack's symptoms is:
26. Obsessive-compulsive disorder (OCD)

Here's my step-by-step reasoning:
1. **Intrusive thoughts**: Jack experiences recurring, unwanted images of touching his nieces and nephews in a sexual manner. This is a classic symptom of OCD, where individuals experience intrusive thoughts, urges, or images that are distressing and repetitive.

2. **Lack of desire or arousal**: Jack explicitly states that he has no desire to touch the children and does not experience any sexual arousal during these images. This rules out pedophilia (option 30) and other paraphilic disorders.

3. **Excessive worry and anxiety**: Jack is significantly distressed by these thoughts and worries about "what if" he were to act on them. This excessive worry and anxiety are characteristic of OCD.

4. **Avoidance behavior**: Jack starts avoiding his nieces and nephews and refuses to spend time alone with them due to his fear of acting on his unwanted thoughts. This avoidance behavior is a common coping mechanism for individuals with OCD.

5. **Duration and distress**: The symptoms have been present for more than five years and cause Jack significant distress, which meets the criteria for OCD.

Ranking my choices in order of likelihood:
1. Obsessive-compulsive disorder (OCD)
2. Generalized anxiety disorder (option 18) - Although Jack's symptoms are primarily focused on intrusive thoughts and avoidance, there may be some overlap with generalized anxiety disorder. However, the specificity of his symptoms and the presence of intrusive thoughts make OCD a more likely diagnosis.

I did not consider other options, such as adjustment disorder or specific phobia, as they do not fully capture the complexity of Jack's symptoms, particularly the presence of intrusive thoughts and excessive worry.

V3 (2022). Gemini pro: N/A

Vignette 4 (2022)

Unless otherwise stated, Jack's symptoms below are not because of another psychology condition. They are not due to a general medical condition. They are not due to substance use. The symptoms, unless stated, have been present for more than five years and cause him significant distress.

Jack, a middle-aged, highly religious man, believes that one should not have any negative thoughts about religion. He greatly worries when he notices himself having such negative religious thoughts (i.e. why does God have bad things happen to good people?). When these "bad" thoughts occur, as they frequently do, he becomes distressed and fears God will punish him. Jack then prays repeatedly to himself until he feels safe from harm, this can go on for hours. He knows that his thoughts come from within his own mind and are excessive in nature. However, even knowing this he remains upset by the thoughts and is not able to stop them.

Based on the vignette which option most likely applies? If more than one, please mark all options that may apply. If you marked more than one of the above, please rank your choices in order of likelihood.
Lastly, use behavioral signs and symptoms from the vignette to step by step deduce the correct response and show me your reasoning for your choice of most likely option.

1. Adjustment disorder
2. Agoraphobia with panic disorder
3. Agoraphobia without panic disorder
4. Alcohol abuse
5. Alcohol dependence
6. Anorexia nervosa
7. Antisocial personality disorder
8. Asperger's disorder
9. Attention deficit hyperactivity disorder
10. Autism disorder
11. Avoidant personality disorder
12. Bipolar I disorder
13. Bipolar II disorder
14. Body dysmorphic disorder
15. Borderline personality disorder
16. Bulimia Nervosa
17. Delusional disorder
18. Generalized anxiety disorder
19. Histrionic personality disorder
20. Hypochondriasis
21. Impulse control disorder, NOS
22. Major depression disorder
23. Marital problems
24. Narcissistic personality disorder
25. No disorder/condition
26. Obsessive-compulsive disorder
27. Obsessive-compulsive personality disorder
28. Panic disorder
29. Paranoid personality disorder
30. Pedophilia
31. Posttraumatic stress disorder
32. Primary Insomnia
33. Psychosis
34. Sexual identity confusion
35. Schizophrenia
36. Schizoid personality disorder
37. Schizotypal personality disorder
38. Social phobia/Social anxiety disorder
39. Somatization disorder
40. Specific phobia
41. Strong religious values
42. Tourette's
43. Trichotillomania
44. Other (specify)

LLM responses to vignette 4 (2022)

V4 (2022). ChatGPT-4

Obsessive-compulsive disorder (OCD) (Option 26).

Here's the step-by-step reasoning for this conclusion:

1. **Presence of Obsessions**: Jack has persistent, unwanted thoughts about negative religious ideas which cause him distress. This fits the definition of obsessions as seen in OCD, which are intrusive and unwanted thoughts that cause marked anxiety or distress.
2. **Presence of Compulsions**: To neutralize these thoughts, Jack engages in compulsive behavior – specifically, he prays repeatedly until he feels safe. This is a typical compulsion, which is a behavior or mental act that a person feels driven to perform in response to an obsession.
3. **Recognition of Excessiveness**: Jack is aware that his thoughts are excessive and come from his own mind, which aligns with OCD where individuals can recognize that their obsessions and compulsions are unreasonable or excessive.
4. **Duration and Distress**: The symptoms have been present for more than five years and cause significant distress, fulfilling the chronicity and impact criteria often required for a diagnosis of OCD.
5. **Exclusion of Other Factors**: The information rules out a general medical condition, substance use, or another psychological condition as the cause of his symptoms, focusing the diagnosis on a mental health disorder that is primary and not secondary to another condition.

Other options are less likely based on the vignette:

- **Adjustment disorder** typically follows a significant life event or change and does not involve the specific pattern of obsessions and compulsions.
- **Agoraphobia** and **Panic disorder** are characterized by fear and avoidance of places or situations where escape might be difficult, not religiously themed obsessions and compulsions.
- **Substance-related disorders** are ruled out by the information provided.
- **Eating disorders** like Anorexia Nervosa and Bulimia Nervosa are not suggested by any of the behaviors described.
- **Personality disorders** manifest in enduring patterns of perceiving, relating to, and thinking about the environment and oneself that are not described in the vignette.
- **Mood disorders** like Bipolar Disorder and Major Depression Disorder do not include the specific obsessions and compulsive behaviors.
- **Anxiety disorders** like Generalized Anxiety Disorder may involve worry, but not the specific compulsive behaviors seen in OCD.
- **Somatoform disorders** involve physical symptoms without a medical cause, which is not the case here.
- **Psychotic disorders** such as Schizophrenia involve a range of symptoms like hallucinations and delusions that are not mentioned in Jack's case.
- **Obsessive-compulsive personality disorder** is about a general pattern of concern with orderliness, perfectionism, and control rather than specific unwanted obsessions and compulsions that cause distress.

Therefore, Obsessive-compulsive disorder (Option 26) is the most fitting diagnosis based on the information given.

V4 (2022). Llama 3

Based on the vignette, I believe the most likely option is:
26. Obsessive-compulsive disorder

Here's my step-by-step reasoning:
1. Jack experiences recurring, unwanted thoughts (negative religious thoughts) that cause him significant distress.

2. He recognizes that these thoughts come from within his own mind and are excessive in nature, which suggests insight into the irrationality of his thoughts.

3. Despite this insight, he is unable to stop or control these thoughts, which is a hallmark feature of obsessive-compulsive disorder (OCD).

4. Jack engages in repetitive behavior (praying repeatedly to himself) to reduce his anxiety and perceived risk of punishment from God. This behavior is excessive and time-consuming (hours), which is another key feature of OCD.

5. The presence of both obsessions (unwanted thoughts) and compulsions (repetitive behavior) in response to those obsessions strongly suggests OCD.

Ranking my choices in order of likelihood:
1. 26. Obsessive-compulsive disorder
2. 18. Generalized anxiety disorder (although the specific features of OCD are more prominent in the vignette)

I did not choose other options because:
* Adjustment disorder (1) would require a specific stressor or event, which is not mentioned in the vignette.
* Agoraphobia with or without panic disorder (2-3) does not fit, as there is no mention of fear or avoidance of specific situations or places.
* There is no indication of substance abuse or dependence (4-5), eating disorders (6, 16), personality disorders (7, 11, 15, 19, 24, 27, 29, 36, 37), or other conditions listed.

The presence of strong religious values (41) is a relevant aspect of Jack's background but does not constitute a psychological disorder in itself.

V4 (2022). Gemini pro

Analyzing Jack's symptoms:

Based on the information provided, here's a step-by-step analysis of Jack's symptoms and the most likely diagnoses:

1. Symptoms:
   - Obsessive thoughts: Jack experiences frequent negative thoughts about religion, causing him significant distress.
   - Compulsive behavior: He engages in repetitive praying for hours to alleviate his anxiety and fear of punishment.
   - Insight: He recognizes that his thoughts are excessive and originate from within him.
   - Religious beliefs: His distress stems from his strong religious beliefs and the fear of divine retribution.

2. Ruling out other options:
   - Other psychological conditions: The prompt specifies that the symptoms are not due to another psychological condition.
   - General medical conditions and substance use: The prompt also excludes these factors.
   - Timeframe: The symptoms have been present for over five years, making transient conditions like adjustment disorder less likely.

3. Potential diagnoses:
Based on the symptoms and information provided, the following diagnoses are most likely:

- Obsessive-compulsive disorder (OCD): Jack's recurrent intrusive thoughts and compulsive praying strongly align with OCD criteria. His awareness of the excessive nature of his thoughts further supports this diagnosis.
- Scrupulosity OCD: This subtype of OCD specifically focuses on religious and moral obsessions and compulsions, making it highly relevant to Jack's case.
- Generalized anxiety disorder (GAD): Jack's constant worry and fear about his thoughts and potential punishment could also indicate GAD.

4. Ranking the options:
   1. Obsessive-compulsive disorder (OCD), specifically Scrupulosity OCD: This is the most likely diagnosis given the prominent presence of intrusive thoughts, compulsive behaviors, and religious context.
   2. Generalized anxiety disorder (GAD): This is a secondary possibility due to the underlying anxiety and worry Jack experiences.

5. Behavioral signs and symptoms supporting the diagnosis:
   - Obsessive thoughts: Jack's frequent negative thoughts about religion.
   - Compulsive behavior: His repetitive praying for extended periods.
   - Fear of punishment: His anxiety and distress stem from the belief that God will punish him for his thoughts.
   - Insight: His awareness that his thoughts are excessive and originate from within him.
   - Religious context: The content of his obsessions and compulsions is directly related to his religious beliefs.

6. Conclusion:
Based on the analysis, Jack's symptoms most likely indicate Obsessive-compulsive disorder (OCD), specifically Scrupulosity OCD. The presence of intrusive thoughts, compulsions, and the

religious context strongly support this diagnosis. While Generalized anxiety disorder might also contribute to his distress, OCD appears to be the primary concern.

Vignette 5 (2022)

Unless otherwise stated, Jack's symptoms below are not because of another psychology condition. They are not due to a general medical condition. They are not due to substance use. The symptoms, unless stated, have been present for more than five years and cause him significant distress.

Jack, a middle-aged man, constantly worries about dirt and germs. He knows that his thoughts come from within his own mind and are excessive in nature. However, even knowing this he remains upset by the thoughts and is not able to stop them. He is unable to complete many of his daily activities because he tries at all costs to avoid touching things he thinks may be dirty. However, if he does touch a "dirty" object, Jack will immediately wash his hands so that he will not catch a disease.

Based on the vignette which option most likely applies? If more than one, please mark all options that may apply. If you marked more than one of the above, please rank your choices in order of likelihood.
Lastly, use behavioral signs and symptoms from the vignette to step by step deduce the correct response and show me your reasoning for your choice of most likely option.

1. Adjustment disorder
2. Agoraphobia with panic disorder
3. Agoraphobia without panic disorder
4. Alcohol abuse
5. Alcohol dependence
6. Anorexia nervosa
7. Antisocial personality disorder
8. Asperger's disorder
9. Attention deficit hyperactivity disorder
10. Autism disorder
11. Avoidant personality disorder
12. Bipolar I disorder
13. Bipolar II disorder
14. Body dysmorphic disorder
15. Borderline personality disorder
16. Bulimia Nervosa
17. Delusional disorder
18. Generalized anxiety disorder
19. Histrionic personality disorder
20. Hypochondriasis
21. Impulse control disorder, NOS
22. Major depression disorder
23. Marital problems
24. Narcissistic personality disorder
25. No disorder/condition

26. Obsessive-compulsive disorder
27. Obsessive-compulsive personality disorder
28. Panic disorder
29. Paranoid personality disorder
30. Pedophilia
31. Posttraumatic stress disorder
32. Primary Insomnia
33. Psychosis
34. Sexual identity confusion
35. Schizophrenia
36. Schizoid personality disorder
37. Schizotypal personality disorder
38. Social phobia/Social anxiety disorder
39. Somatization disorder
40. Specific phobia
41. Strong religious values
42. Tourette's
43. Trichotillomania
44. Other (specify)

LLM responses to vignette 5 (2022)

V5 (2022). ChatGPT-4

The information presented in the vignette points towards a diagnosis of Obsessive-Compulsive Disorder (OCD). Let's walk through the reasoning:

1. Jack exhibits a persistent worry about dirt and germs, which aligns with the obsessions in OCD — unwanted and distressing thoughts.
2. He recognizes these thoughts as his own and excessive, which is typical of OCD where the individual is usually aware of the irrational nature of their obsessions.
3. Despite this awareness, he cannot stop these thoughts; this is characteristic of the compulsive aspect of OCD, where the individual feels driven to perform certain behaviors or rituals.
4. Jack's need to wash his hands after touching an object he considers "dirty" is a classic compulsive behavior seen in OCD, aimed at reducing the distress caused by obsessions.
5. The symptoms are causing significant distress and impairment in his daily activities, another key component of OCD diagnosis.
6. The symptoms have been present for more than five years, suggesting a chronic condition, which fits the profile of OCD.

The other conditions listed either do not align with the obsessive and compulsive symptoms described (such as agoraphobia, substance use disorders, mood disorders, eating disorders, personality disorders, and other psychiatric conditions) or lack supporting evidence in the vignette.

Therefore, the most likely diagnosis based on the vignette is:

26. Obsessive-compulsive disorder

This choice is made based on the presence of obsessions (persistent worries about dirt and germs) and compulsions (hand washing after touching a "dirty" object), the recognition by Jack of these thoughts as excessive, the chronicity of the condition, and the significant distress and functional impairment it causes.

V5 (2022). Llama 3

Based on the vignette, the most likely option that applies to Jack is:
26. Obsessive-compulsive disorder (OCD)

Here's my step-by-step reasoning:
1. **Excessive and persistent worries**: Jack constantly worries about dirt and germs, which is an excessive and persistent concern that causes him significant distress.

2. **Recognition of thoughts as excessive**: Jack knows that his thoughts come from within his own mind and are excessive in nature, which suggests insight into his symptoms.

3. **Avoidance behaviors**: Jack tries to avoid touching things he thinks may be dirty, which is a common avoidance behavior seen in OCD.

4. **Compulsive behavior**: When Jack does touch a "dirty" object, he immediately washes his hands to prevent catching a disease, which is a compulsive behavior aimed at reducing anxiety.

These behavioral signs and symptoms are consistent with the diagnostic criteria for Obsessive-Compulsive Disorder (OCD). The presence of obsessive thoughts (worries about dirt and germs), recognition of their excessiveness, and compulsive behaviors (hand washing) strongly support this diagnosis.

Other options that could be considered but are less likely:
* 18. Generalized anxiety disorder: While Jack's worries about dirt and germs are excessive, they are focused on a specific theme rather than being more general, which is more characteristic of GAD.
* 27. Obsessive-compulsive personality disorder: While Jack's symptoms share some similarities with OCPD, his symptoms are more severe and interfere with his daily activities, which is more consistent with OCD.

Ranking my choices in order of likelihood:
1. 26. Obsessive-compulsive disorder
2. 18. Generalized anxiety disorder
3. 27. Obsessive-compulsive personality disorder

V5 (2022). Gemini pro

Applying the vignette to the DSM-5 criteria for potential diagnoses:

Based on the information provided, several options could potentially apply to Jack's situation. Here's a breakdown of the most likely diagnoses, ranked in order of likelihood:

1. Obsessive-Compulsive Disorder (OCD):
   - Strong evidence:
     - Obsessions: Jack constantly worries about dirt and germs, recognizing these thoughts as excessive.
     - Compulsions: He engages in handwashing to alleviate anxiety caused by the contamination fears.
     - Significant distress and impairment: His daily life is significantly impacted due to his attempts to avoid "dirty" objects and the time spent washing his hands.
     - Duration: Symptoms have persisted for over five years.

2. Specific Phobia (Germs):
   - Supporting evidence:
     - Intense fear and anxiety: Jack exhibits a strong aversion towards germs, fearing contamination and potential illness.
     - Avoidance: He actively avoids contact with objects he perceives as contaminated.
     - Distress and impairment: His avoidance behaviors significantly impact daily functioning.
     - Duration: Symptoms have been present for over five years.
   - Less likely than OCD: The presence of compulsions (handwashing) suggests a stronger case for OCD.

3. Hypochondriasis:
   - Possible evidence: Preoccupation with the fear of contracting a disease due to contamination.
   - Less likely than OCD and Specific Phobia: The primary focus seems to be on avoiding contamination rather than fearing illness itself. Additionally, handwashing serves to prevent contamination rather than alleviate anxiety about illness, as seen in hypochondriasis.

4. Social Anxiety Disorder (SAD):
   - Limited evidence: The vignette doesn't explicitly mention social situations or fear of scrutiny. While the fear of germs might lead to avoidance of specific social settings (e.g., public restrooms), the core anxieties seem to revolve around contamination rather than social judgment.

Other listed diagnoses are unlikely due to a lack of supporting evidence in the vignette or the presence of features that contradict the criteria for those diagnoses.

Conclusion:

Based on the analysis, Obsessive-Compulsive Disorder (OCD) appears to be the most likely diagnosis for Jack, followed by Specific Phobia (Germs) as a secondary possibility.

Reasoning:

The presence of both obsessions (intrusive thoughts about contamination) and compulsions (handwashing to alleviate anxiety) strongly suggests OCD. The significant distress and impairment caused by these symptoms further support this diagnosis. While Jack's fear of germs also aligns with Specific Phobia, the presence of compulsions points more towards OCD.

It's important to note that a definitive diagnosis should be made by a qualified mental health professional through a comprehensive evaluation.

Vignette 6 (2022)

Unless otherwise stated, Jack’s symptoms below are not because of another psychology condition. They are not due to a general medical condition. They are not due to substance use. The symptoms, unless stated, have been present for more than five years and cause him significant distress.

Jack, a middle-aged man, worries when things are not orderly and systematic. He becomes anxious when individuals move his belongings and feels he must immediately return the objects to their proper place. He also rearranges things that are not in order to place them how they “should be.” When things are not in proper order Jack is unable to focus until the objects are back in their correct place. He knows that his thoughts come from within his own mind and are excessive in nature. However, even knowing this he remains upset by the thoughts and is not able to stop them.

Based on the vignette which option most likely applies? If more than one, please mark all options that may apply. If you marked more than one of the above, please rank your choices in order of likelihood.
Lastly, use behavioral signs and symptoms from the vignette to step by step deduce the correct response and show me your reasoning for your choice of most likely option.

1. Adjustment disorder
2. Agoraphobia with panic disorder
3. Agoraphobia without panic disorder
4. Alcohol abuse
5. Alcohol dependence
6. Anorexia nervosa
7. Antisocial personality disorder
8. Asperger’s disorder
9. Attention deficit hyperactivity disorder
10. Autism disorder
11. Avoidant personality disorder
12. Bipolar I disorder
13. Bipolar II disorder
14. Body dysmorphic disorder
15. Borderline personality disorder
16. Bulimia Nervosa

17. Delusional disorder
18. Generalized anxiety disorder
19. Histrionic personality disorder
20. Hypochondriasis
21. Impulse control disorder, NOS
22. Major depression disorder
23. Marital problems
24. Narcissistic personality disorder
25. No disorder/condition
26. Obsessive-compulsive disorder
27. Obsessive-compulsive personality disorder
28. Panic disorder
29. Paranoid personality disorder
30. Pedophilia
31. Posttraumatic stress disorder
32. Primary Insomnia
33. Psychosis
34. Sexual identity confusion
35. Schizophrenia
36. Schizoid personality disorder
37. Schizotypal personality disorder
38. Social phobia/Social anxiety disorder
39. Somatization disorder
40. Specific phobia
41. Strong religious values
42. Tourette's
43. Trichotillomania
44. Other (specify)

LLM responses to vignette 6 (2022)

V6 (2022). ChatGPT-4

The description in the vignette suggests that Jack exhibits behaviors consistent with Obsessive-Compulsive Disorder (OCD) or Obsessive-Compulsive Personality Disorder (OCPD). Both conditions involve an obsession with orderliness and perfectionism. However, they differ in the nature of their symptoms and the distress they cause.

The key points from the vignette are:

- Jack worries when things are not orderly and systematic.
- He becomes anxious when things are moved from their place.
- He feels compelled to rearrange things to where they "should be."
- He experiences significant distress from these thoughts.
- He is aware that his thoughts are excessive.
- He is unable to stop these thoughts even though he recognizes them as coming from within and as being excessive.

From these points, we can deduce the following:

1. Adjustment Disorder: This typically occurs in response to a significant life change or stressor, which is not mentioned in the vignette.
2. Agoraphobia (with or without panic disorder): This involves fear and avoidance of places or situations that might cause panic, helplessness, or embarrassment. The vignette does not mention avoidance of places or situations due to these fears.
3. Alcohol Abuse/Dependence: There is no mention of alcohol use or related problems.
4. Eating Disorders (Anorexia Nervosa, Bulimia Nervosa): The vignette does not mention any symptoms related to eating disorders.
5. Personality Disorders (Antisocial, Avoidant, Borderline, Histrionic, Narcissistic, Paranoid, Schizoid, Schizotypal): These disorders have distinct patterns of behavior and inner experiences that do not align with Jack's symptoms as described.
6. Autism Spectrum Disorders (Asperger's, Autism): These are characterized by challenges in social interaction and communication, which is not the focus of Jack's symptoms.
7. Mood Disorders (Bipolar I and II, Major Depression): The symptoms described do not include mood swings or episodes of mania or depression.
8. Body Dysmorphic Disorder: This involves preoccupation with perceived flaws in physical appearance, which is not mentioned in the vignette.
9. Delusional Disorder, Psychosis, Schizophrenia: These conditions involve a break from reality which is not indicated in the vignette.
10. Generalized Anxiety Disorder: While Jack does experience anxiety, it is specifically related to orderliness and not general in nature.
11. Hypochondriasis: This involves preoccupation with having a serious illness, which is not mentioned.
12. Impulse Control Disorders: Jack's behaviors are not impulsive but rather driven by an obsession with order.
13. Posttraumatic Stress Disorder: There is no mention of a traumatic event.
14. Primary Insomnia: Sleep issues are not discussed.
15. Social Phobia/Social Anxiety Disorder: Jack's distress is related to orderliness, not social situations.
16. Somatization Disorder: No mention of experiencing multiple, current, physical symptoms.
17. Specific Phobia: The fear does not pertain to specific objects or situations but rather a general need for order.
18. Strong Religious Values: The vignette does not attribute Jack's behaviors to religious beliefs.
19. Tourette's, Trichotillomania: These involve motor or vocal tics or hair-pulling, which are not mentioned.

Based on this analysis, we can rule out most of the options, focusing on those related to Jack's need for order and resulting distress:

26. Obsessive-Compulsive Disorder (OCD): This is characterized by the presence of obsessions (intrusive, unwanted thoughts that cause distress) and compulsions (repetitive behaviors that the person feels driven to perform in response to an obsession). Jack's need to have things orderly and his distress when they are not, along with his inability to control these thoughts, is characteristic of OCD.
27. Obsessive-Compulsive Personality Disorder (OCPD): This disorder involves a preoccupation with orderliness, perfectionism, and control. Jack's distress and the chronic nature of his symptoms could suggest OCPD. However, OCPD typically does

not involve the recognition that the behaviors are excessive, which Jack does acknowledge.

In ranking these, OCD seems more likely because Jack has insight into his condition and recognizes his thoughts as excessive. People with OCPD often do not have this insight. Therefore, I would rank the likelihood as follows:

1. Obsessive-Compulsive Disorder (OCD)
2. Obsessive-Compulsive Personality Disorder (OCPD)

Jack's recognition of his thoughts as excessive and his distress suggest OCD is the more likely diagnosis.

V6 (2022). Llama 3

Based on the vignette, I believe the most likely option is:
26. Obsessive-compulsive disorder

Here's my step-by-step reasoning:
1. Jack's symptoms revolve around a need for order and symmetry, which is a common theme in obsessive-compulsive disorder (OCD).

2. He becomes anxious when things are out of place and feels an overwhelming urge to correct them, which suggests a strong emotional response to perceived imperfections.

3. Jack rearranges objects to put them in their "proper" place, which is a classic behavioral symptom of OCD.

4. His inability to focus until things are in order indicates that his obsessions and compulsions interfere with his daily functioning.

5. The fact that Jack recognizes his thoughts as excessive and coming from within his own mind, but still can't stop them, suggests a level of insight and self-awareness that is often seen in individuals with OCD.

While Jack's symptoms also share some similarities with obsessive-compulsive personality disorder (OCPD), the presence of clear anxiety and distress in response to perceived imperfections points more towards OCD.

Ranking my choices in order of likelihood:
1. 26. Obsessive-compulsive disorder
2. 27. Obsessive-compulsive personality disorder (less likely due to the presence of anxiety and distress)

I did not consider other options because they do not align with the specific symptoms described in the vignette.

V6 (2022). Gemini pro

Applying the DSM-5 criteria to Jack's symptoms:

Based on the information provided, Jack's symptoms most likely point to Obsessive-Compulsive Disorder (OCD). Here's a breakdown of the reasoning:

1. Presence of obsessions and compulsions:

- Obsessions: Jack experiences intrusive thoughts about the need for order and symmetry, causing him significant distress. He recognizes these thoughts as excessive but feels unable to control them. This aligns with the DSM-5 criteria for obsessions.
- Compulsions: Jack engages in repetitive behaviors like rearranging objects and returning them to their "proper" place. He feels compelled to perform these actions to alleviate the anxiety caused by the obsessions. This aligns with the DSM-5 criteria for compulsions.

2. Timeframe and distress:

- The symptoms have been present for more than five years, exceeding the minimum duration required for an OCD diagnosis.
- Jack experiences significant distress due to his obsessions and compulsions, impacting his daily life and causing emotional suffering.

3. Absence of other explanations:

- The vignette specifies that Jack's symptoms are not due to another psychological condition, a general medical condition, or substance use.

Differential Diagnoses:
While OCD is the most likely diagnosis, other conditions share some overlapping features. Here's a comparison:

- Obsessive-Compulsive Personality Disorder (OCPD): Individuals with OCPD also have a strong need for order and control, but their focus is on perfectionism and following rules rather than intrusive thoughts. Additionally, the distress caused by OCPD is often less severe than in OCD.
- Generalized Anxiety Disorder (GAD): People with GAD experience excessive worry and anxiety about various aspects of life, but they don't typically have the specific obsessions and compulsions seen in OCD.
- Body Dysmorphic Disorder (BDD): Individuals with BDD are preoccupied with perceived flaws in their appearance, leading to compulsive behaviors like excessive grooming or mirror checking. However, the focus of their concerns is on their physical appearance, not on order and symmetry as in Jack's case.

Likelihood Ranking:

1. Obsessive-Compulsive Disorder (OCD): High likelihood due to the presence of both obsessions and compulsions causing significant distress.
2. Obsessive-Compulsive Personality Disorder (OCPD): Possible, but less likely as the focus is more on perfectionism and rules rather than intrusive thoughts, and the distress is typically less severe.

3. Generalized Anxiety Disorder (GAD): Possible, but less likely as the specific obsessions and compulsions are not typically present in GAD.
4. Body Dysmorphic Disorder (BDD): Unlikely, as the focus of Jack's concerns is on order and symmetry, not on perceived flaws in his appearance.

Conclusion:
Based on the information provided, Obsessive-Compulsive Disorder (OCD) is the most likely diagnosis for Jack. However, a comprehensive evaluation by a mental health professional is recommended for a definitive diagnosis and appropriate treatment plan.

2. Control vignettes and LLMs responses

1.Major depression

https://www.ncbi.nlm.nih.gov/pmc/articles/PMC6220621/

“A 28-year-old married, employed female computer programmer with two young children (one aged four years, the other aged nine months) is seen for a four-week history of fatigue, insomnia, headache, abdominal discomfort, and difficulty concentrating at work. She denies signs and symptoms of an acute infectious process and did not have headache or abdominal pain before the previous month. She is breastfeeding. She has obtained intermittent relief from headache by using acetaminophen, and she takes a multivitamin regularly. Normal menses has resumed. She is appropriately and professionally dressed, and her children accompany her in

the examination room. She appears tired but in no acute distress. Results of physical examination, including neurologic screening, are normal.

In addition to sleep disturbance, decreased energy, and difficulty concentrating, the patient in the above example admitted being sad and tearful as well as feeling guilty and worrying about her parenting skills, and she had lost interest in socializing. She also admitted to worrying about work performance and being somewhat irritable with her husband.”

What do you think are the three most likely medical diseases or diagnoses that could be causing this health problem? Please rank your choices in order of likelihood.

2.Generalized Anxiety Disorder (GAD)

Source: chrome-extension://efaidnbmnnnibpcajpcglclefindmkaj/https://www.nice.org.uk/guidance/cg113/resources/clinical-case-scenarios-pdf-136292509.

Mary is aged 42 years, divorced with two children, employed part time and cares for her mother who has Alzheimer’s disease.

Mary has no significant past medical history, although she frequently makes appointments with her GP and practice nurse about problems experienced by her and her children. She was moderately depressed following her divorce 5 years ago and was offered antidepressants but

declined them. She was referred for six sessions of counselling, which led to some improvement in her symptoms.

Mary complains of feeling ‘stressed’ all the time and constantly worries about ‘anything and everything’. She describes herself as always having been a ‘worrier’ but her anxiety has become much worse in the past 12 months since her mother became unwell, and she no longer feels that she can control these thoughts. When worried, Mary feels tension in her shoulders, stomach and legs, her heart races and sometimes she finds it difficult to breathe. Her sleep is poor with difficulty getting off to sleep due to worrying and frequent wakening. She feels tired and irritable. She does not drink any alcohol.

What do you think are the three most likely medical diseases or diagnoses that could be causing this health problem? Please rank your choices in order of likelihood.

3.PTSD

https://div12.org/case_study/victor-ptsd/

Victor is a 27-year-old man who comes to you for help at the urging of his fiancée. He was an infantryman with a local Marine Reserve unit who was honorably discharged in 2014 after serving two tours of duty in Iraq. His fiancé has told him he has “not been the same” since his second tour of duty and it is impacting their relationship. Although he offers few details, upon questioning he reports that he has significant difficulty sleeping, that he “sleeps with one eye open” and, on the occasions when he falls into a deeper sleep, he has nightmares. He endorses experiencing several traumatic events during his second tour, but is unwilling to provide specific

details – he tells you he has never spoken with anyone about them and he is not sure he ever will. He spends much of his time alone because he feels irritable and doesn't want to snap at people. He reports to you that he finds it difficult to perform his duties as a security guard because it is boring and gives him too much time to think. At the same time, he is easily startled by noise and motion and spends excessive time searching for threats that are never confirmed both when on duty and at home. He describes having intrusive memories about his traumatic experiences on a daily basis but he declines to share any details. He also avoids seeing friends from his Reserve unit because seeing them reminds him of experiences that he does not want to remember.

SYMPTOMS

- Hypervigilance
- Intrusive Thoughts
- Irritability
- Loss of Interest
- Sleep Difficulties
- Trauma

What do you think are the three most likely medical diseases or diagnoses that could be causing this health problem? Please rank your choices in order of likelihood.

4. MDD in an adolescent

https://onlinelibrary.wiley.com/doi/10.1002/da.20457

The patient was a 15-year-old Puerto Rican adolescent female living with both her parents and a younger sibling. Her parents presented with significant marital problems had been separated several times and were discussing divorce. Her mother reported having a history of psychiatric treatment for depression and anxiety and indicated that the patient's father suffered from bipolar disorder and had been receiving psychiatric treatment. He was hospitalized on multiple occasions during previous years for serious psychiatric symptoms.

The patient was failing several classes in school, and her family was in the process of looking for a new school due to her failing grades and difficulties getting along with her classmates. She presented the following symptoms: frequent sadness and crying, increased appetite and overeating, guilt, low self-concept, anxiety, irritability, insomnia, hopelessness, and difficulty concentrating. In addition, she presented difficulties in her interpersonal relationships, persistent negative thoughts about her appearance and scholastic abilities, as well as guilt regarding her parents' marital problems. The patient's medical history revealed that she suffered from asthma, used eyeglasses, and was overweight.

What do you think are the three most likely medical diseases or diagnoses that could be causing this health problem?

5. uni- or bipolar depression (both correct)

https://focus.psychiatryonline.org/doi/10.1176/appi.focus.20150047

“I’ve been struggling with this depression for months, and nothing seems to be helping this time around,” the patient said when she first met you. Brenda reported two previous lifetime episodes of major depression, both with clear remissions: one at age 20 during her sophomore year in college at a large university (remitted with cognitive-behavioral therapy provided through campus counseling services) and a second at age 26 (remitted with citalopram from her primary

care physician). “This time, it began with trouble sleeping, like it always does,” she said, “and then there was the anxiety, the pacing and fidgeting, and the crying.” She further revealed that she was experiencing decreased appetite and enough weight loss that her clothing all “felt baggy.” In your interview, she reported feeling “like I’m a loser, with this damn depression coming back again,” but denied having feelings of guilt. She acknowledged that she could still enjoy getting together with friends for an activity, “but the good feelings fade fast—a few hours later and I’m back down in the dumps.” She noted that she felt fatigued most days, “but with only a few hours of sleep a night, who wouldn’t?” She denied having suicidal thoughts or plans, adding, “I had a friend in college who overdosed on some pills, and she ended up needing a liver transplant. I would never want to inflict that on my family and friends.”

At this point in the assessment of the patient’s history, your differential diagnosis includes current? Please rank your choices in order of likelihood.

- Unipolar major depressive disorder
- Depression as a part of bipolar disorder
- Attention-deficit hyperactivity disorder
- Substance-induced mood disorder
- Generalized anxiety disorder

6. Social anxiety vignette

https://psycnet.apa.org/record/2017-27521-001

Presents a case vignette of a 26-year-old student who reported feeling very anxious when giving a presentation, taking an examination, or meeting an authority figure. In these situations, he has palpitations, tremors, blushing, and sweating, and he is fearful that he will embarrass himself. He reported having few social contacts and avoids going to parties and making phone calls, but he feels lonely. His anxieties started during his teenage years and have increased considerably since he started attending a university. The patient described in the vignette reported having social fears when giving a presentation, taking an examination, or meeting an

authority figure. These fears were associated with palpitations, tremors, blushing, and sweating. He also reported avoidance of social contact.

What do you think are the three most likely medical diseases or diagnoses that could be causing this health problem?

7. Panic disorder

https://div12.org/case_study/dave-panic-disorder/

Dave is a 41-year-old male who was referred by his primary care physician after presenting to the ER with difficulty breathing. Dave's physician was unable to find a medical explanation for his symptoms, which left Dave feeling confused, stressed, and angry. Over the last 6 months, Dave has had several instances where he felt an intense fear that would reach a peak within a

few minutes. During these instances, he would also experience sweating, heart palpitations, chest pain and discomfort, and shortness of breath. At times, Dave worried that might die. As a result, Dave has persistent worry about having another attack. In addition, he has begun to avoid unfamiliar places and people where it may be difficult to get help in the event of another panic attack. The panic and associated avoidance are significantly impacting Dave’s life as he has been turning down social invitations, making excuses to stay at home whenever possible, and relying on his wife to drive their children to their various activities. Although she was understanding at first, Dave’s wife has grown frustrated with what she perceives as his irrational fear of panic attacks.

What do you think are the three most likely medical diseases or diagnoses that could be causing this health problem?

Responses of chatbots to control vignettes 1. Major depression

ChatGPT-4

The symptoms described for the 28-year-old female computer programmer suggest a few potential medical issues, which can be ranked in order of likelihood as follows:

1. **Postpartum Depression**: The timing of the symptoms relative to her recent pregnancy, her emotional symptoms including sadness, tearfulness, feelings of guilt regarding her parenting skills, and irritability, in addition to physical symptoms such as fatigue and insomnia, strongly point towards postpartum depression. This condition is common in the postpartum period and could explain both her physical and psychological symptoms.
2. **Generalized Anxiety Disorder (GAD)**: Her worry about her performance at work, parenting skills, and the symptoms of irritability, difficulty concentrating, and sleep disturbances could also indicate GAD. Anxiety disorders can cause a wide range of physical symptoms, including headaches and abdominal discomfort.
3. **Thyroid Dysfunction (e.g., Hypothyroidism)**: Given her fatigue, difficulty concentrating, and headaches, a thyroid function test could be useful. Thyroid issues are common in postpartum women and can cause a variety of symptoms that impact both physical and mental health.

These diagnoses are not mutually exclusive and could coexist, amplifying her symptoms. Medical evaluation including a thorough psychological assessment and possibly blood tests (like thyroid function tests) would be necessary to clarify her diagnosis and guide treatment.

Gemini Pro

Based on the information provided, the three most likely medical diagnoses for this patient, in order of likelihood, are:

1. Postpartum Depression (PPD)

- **Reasoning:**
  - The patient's symptoms including fatigue, insomnia, headache, abdominal discomfort, difficulty concentrating, sadness, tearfulness, guilt, worry, loss of interest in socializing, and irritability are highly suggestive of PPD.
  - The timing of the onset of symptoms within four weeks further strengthens this possibility as PPD typically occurs within the first year following childbirth.
  - Additionally, the patient's breastfeeding status is a risk factor for PPD.

2. Generalized Anxiety Disorder (GAD)

- **Reasoning:**
  - The patient's symptoms of worry, difficulty concentrating, fatigue, and irritability are also consistent with GAD.
  - While GAD can occur at any time, it is not uncommon for it to develop during periods of significant life stress, such as becoming a new parent.

3. Adjustment Disorder with Mixed Anxiety and Depressed Mood

- **Reasoning:**
  - This diagnosis encompasses a range of emotional and behavioral symptoms that occur in response to a stressful life event, such as childbirth and adjusting to the demands of motherhood.
  - The patient's symptoms of fatigue, insomnia, difficulty concentrating, sadness, tearfulness, worry, and irritability all fall within the spectrum of this disorder.

Important Note

It is crucial to remember that this information should not be used for self-diagnosis or treatment. Only a qualified healthcare professional can make an accurate diagnosis and recommend the appropriate treatment plan.

The patient should be encouraged to consult with a doctor or mental health professional for a comprehensive evaluation and personalized guidance.

Llama 3

Based on the symptoms and information provided, here are my top three likely medical diseases or diagnoses that could be causing this health problem, ranked in order of likelihood:

1. **Postpartum Depression (PPD)**: Given the patient's recent childbirth, breastfeeding, and normal resumption of menses, PPD is a strong possibility. The symptoms of fatigue, insomnia, headache, difficulty concentrating, sadness, tearfulness, guilt, and worry about parenting skills are all consistent with PPD. Additionally, the patient's irritability with her husband and loss of interest in socializing further support this diagnosis.

2. **Anxiety Disorder (Generalized Anxiety Disorder or Adjustment Disorder with Anxiety)**: The patient's symptoms of anxiety, worry, and difficulty concentrating at work suggest an anxiety disorder. The fact that she has obtained intermittent relief from headaches using acetaminophen may indicate that her anxiety is contributing to her physical symptoms. The stress of caring for two young children, working full-time, and adjusting to new motherhood may have triggered an anxiety disorder.

3. **Iron Deficiency Anemia**: Although less likely than the first two options, iron deficiency anemia should be considered given the patient's history of breastfeeding and potential blood loss during childbirth. Fatigue, headaches, and difficulty concentrating can be symptoms of iron deficiency anemia. However, the absence of other typical symptoms like pallor, shortness of breath, or dizziness reduces the likelihood of this diagnosis.

Please note that these are just hypothetical possibilities based on the provided information and should not be considered a definitive diagnosis. A thorough medical evaluation and diagnostic tests would be necessary to determine the underlying cause of the patient's symptoms.

Responses of chatbots to control vignette 2. Generalized Anxiety Disorder (GAD)

ChatGPT-4

Based on the description of Mary's symptoms and her current life circumstances, the three most likely medical conditions or diagnoses are:

1. **Generalized Anxiety Disorder (GAD)**: Mary's constant worry about various aspects of life, difficulty controlling worry, physical symptoms like muscle tension, racing heart, and difficulty breathing, along with sleep disturbances, align closely with the criteria for GAD. Her history of being a 'worrier' that has recently intensified could suggest that her anxiety has evolved into a more pervasive disorder.
2. **Major Depressive Disorder (MDD)**: Although Mary was previously treated for moderate depression following her divorce, some symptoms such as fatigue, irritability, and sleep disturbances suggest that she might be experiencing a relapse or continuation of depressive symptoms. Her significant life stressors, including caring for an ill parent, could exacerbate or trigger depressive episodes.
3. **Chronic Stress**: This is a broader condition but is distinct in its physical and psychological impact. Chronic stress can lead to a variety of symptoms including those Mary is experiencing, such as muscle tension, sleep problems, irritability, and exhaustion. Given her roles as a caregiver and a parent, along with her employment, she may be experiencing ongoing stress without adequate relief or coping strategies.

These diagnoses are not mutually exclusive, and it's possible Mary could be experiencing a combination of them. It would be important for her to consult with her healthcare provider to discuss these symptoms in detail and possibly undergo a formal assessment to receive appropriate treatment.

Gemini Pro

Based on the information provided, here are the three most likely medical diagnoses that could be causing Mary's health problems, ranked in order of likelihood:

1. Generalised Anxiety Disorder (GAD):

- **Symptoms:** Mary describes experiencing constant worry and anxiety about various things, which has worsened in the past year. These worries are accompanied by physical symptoms like tense muscles, rapid heartbeat, and breathing difficulties. She also experiences sleep disturbances, fatigue, and irritability. These symptoms are highly suggestive of GAD, a common mental health disorder characterized by excessive and uncontrollable worry.

2. Adjustment Disorder with Mixed Anxiety and Depressed Mood:

- **Symptoms:** Mary's symptoms emerged following the stressful life event of her mother's illness and her divorce. She describes feeling overwhelmed and unable to cope, which has led to a combination of anxiety and depressed mood. This presentation aligns with Adjustment Disorder, a condition where emotional and behavioral symptoms develop in response to a stressful event.

3. Post-traumatic Stress Disorder (PTSD):

- **Symptoms:** While less likely than the above diagnoses, PTSD should be considered, especially if Mary experienced any traumatic events in her past, including her divorce. Flashbacks, nightmares, and intense emotional reactions to reminders of the trauma could contribute to her anxiety and sleep problems.

Additional Considerations:

- **Physical health conditions:** It's important to rule out any underlying medical conditions that could be contributing to Mary's symptoms, such as thyroid problems or hormonal imbalances.
- **Substance use:** Although Mary reports not drinking alcohol, it's essential to inquire about any other substance use, including caffeine or medications, that might be affecting her sleep and anxiety.

Recommendations:

- **Medical evaluation:** Mary should consult her doctor for a comprehensive evaluation to identify the most likely diagnosis and discuss appropriate treatment options.
- **Psychological assessment:** A mental health professional can conduct a thorough assessment to diagnose Mary's condition and recommend suitable interventions, such as cognitive-behavioral therapy (CBT) or medication management.
- **Support groups:** Joining support groups for caregivers or individuals with anxiety disorders could provide Mary with valuable emotional support and coping strategies.

Disclaimer:
This information should not be considered a definitive diagnosis. Only a qualified healthcare professional can diagnose and recommend treatment for Mary's specific condition.

Llama 3

Based on Mary's symptoms and history, here are my top 3 likely medical diseases or diagnoses that could be causing her health problem, ranked in order of likelihood:

1. **Generalized Anxiety Disorder (GAD)**: This is the most likely diagnosis given Mary's persistent and excessive worry about everyday things, feeling stressed all the time, and physical symptoms such as tension, rapid heartbeat, and difficulty breathing. Her poor sleep quality and feelings of tiredness and irritability are also consistent with GAD.

2. **Adjustment Disorder with Anxious Mood**: This diagnosis is a close second in likelihood. Mary's anxiety symptoms have worsened significantly since her mother's illness, which suggests that she may be struggling to cope with the added stress and responsibility of caregiving. Adjustment disorder is a common diagnosis in individuals who experience significant life changes or stressors.

3. **Depression**: Although Mary declined antidepressants in the past, her history of moderate depression following her divorce and current symptoms of fatigue, irritability, and poor sleep quality make depression a possible diagnosis. However, her primary complaint of excessive worry and anxiety suggests that anxiety may be the more prominent feature of her presentation.

It's essential to note that these diagnoses are not mutually exclusive, and Mary may be experiencing comorbid anxiety and depressive disorders. A thorough clinical evaluation and diagnostic assessment would be necessary to determine the underlying cause of Mary's symptoms.

Responses of chatbots to control vignette 3. PTSD

ChatGPT-4

Based on the symptoms described for Victor, here are the three most likely medical diagnoses, ranked in order of likelihood:

1. **Post-Traumatic Stress Disorder (PTSD)**: Victor's symptoms, including hypervigilance, intrusive thoughts, irritability, nightmares, and avoidance of reminders of trauma, are highly characteristic of PTSD, especially considering his history as an infantryman with traumatic experiences during his military service.
2. **Major Depressive Disorder (MDD)**: The loss of interest, sleep difficulties, and irritability can also be signs of major depression. Although PTSD seems more directly linked to his military experiences, depression is also a common co-occurring condition in individuals with PTSD.
3. **Anxiety Disorders**: While PTSD itself encompasses many anxiety-related symptoms, the general category of anxiety disorders could also be relevant, particularly given his hypervigilance and excessive worry about threats. This could potentially include generalized anxiety disorder or panic disorder, although these are less specific to his trauma history compared to PTSD.

It is important for Victor to be evaluated by a mental health professional who can provide a comprehensive assessment and confirm a diagnosis, enabling appropriate treatment planning.

Gemini Pro

Based on the information provided, here are the three most likely diagnoses for Victor, ranked in order of likelihood:

1. Post-traumatic Stress Disorder (PTSD): This is the most likely diagnosis given Victor's symptoms. He reports experiencing several traumatic events during his second tour, which are now causing him significant distress. His hypervigilance, intrusive thoughts, irritability, sleep difficulties, and avoidance behaviors are all classic symptoms of PTSD. Additionally, his reluctance to discuss his experiences and his desire to avoid reminders of his trauma are also consistent with PTSD.

2. Major Depressive Disorder (MDD): Victor's symptoms of low mood, loss of interest, fatigue, and difficulty sleeping are also suggestive of MDD. It's important to note that PTSD and MDD often co-occur, making it possible for Victor to be diagnosed with both conditions.
3. Generalized Anxiety Disorder (GAD): Victor's constant state of worry and hypervigilance, even in situations that are not objectively dangerous, may also indicate GAD. Additionally, his difficulty concentrating and his tendency to be easily startled are also symptoms of GAD.

Additional Considerations:

- It's important to note that this list is based on the limited information provided and a definitive diagnosis can only be made by a qualified mental health professional after a comprehensive evaluation.
- Other diagnoses that could be considered include Adjustment Disorder with Anxiety and Depressed Mood, Social Anxiety Disorder, and Substance Use Disorder (if Victor is self-medicating with alcohol or drugs to cope with his symptoms).
- It's important to encourage Victor to seek professional help. A mental health professional can provide him with a diagnosis, develop a treatment plan, and help him learn coping skills to manage his symptoms.

Additional Resources:

- National Center for PTSD: https://www.ptsd.va.gov/
- National Institute of Mental Health: https://www.nimh.nih.gov/health/topics/post-traumatic-stress-disorder-ptsd
- Anxiety and Depression Association of America: https://adaa.org/

Please note: I am not a medical professional and cannot provide medical advice. The information provided above should not be used to diagnose or treat any medical condition.

Llama 3

Based on the symptoms presented, I believe the three most likely medical diseases or diagnoses that could be causing Victor's health problem are:

1. **Post-Traumatic Stress Disorder (PTSD)**: This is the most likely diagnosis given the presence of symptoms such as hypervigilance, intrusive thoughts, irritability, sleep difficulties, and avoidance behaviors. The traumatic events experienced during his second tour of duty in Iraq are likely contributing to these symptoms.

2. **Anxiety Disorder**: Victor's excessive startle response, constant searching for threats, and feelings of irritability suggest a high level of anxiety. An anxiety disorder, such as generalized anxiety disorder or anxiety disorder not otherwise specified, could be contributing to his symptoms.

3. **Major Depressive Disorder (MDD)**: Although Victor doesn't explicitly report depressive symptoms, his loss of interest in activities and social withdrawal could indicate underlying depression. Additionally, MDD often co-occurs with PTSD and anxiety disorders.

Ranking these diagnoses in order of likelihood is based on the specific combination and severity of symptoms presented. PTSD is the most likely diagnosis due to the direct correlation between Victor's traumatic experiences and his current symptoms. Anxiety Disorder is the second most likely diagnosis, given the prominent anxiety-related symptoms. Major Depressive Disorder is the third most likely diagnosis, as it may be a comorbid condition contributing to Victor's overall symptom profile.

Responses of chatbots to control vignette 4. MDD in an adolescent

ChatGPT-4

Based on the symptoms described for the 15-year-old patient, here are three potential medical diagnoses that could be considered:

1. **Major Depressive Disorder (MDD)**: The symptoms of frequent sadness, crying, insomnia, hopelessness, low self-esteem, and difficulty concentrating align closely with those of major depression. Given the family history of psychiatric issues and the current stressors from her family environment, this is a plausible diagnosis.
2. **Generalized Anxiety Disorder (GAD)**: The patient's symptoms of anxiety, irritability, and difficulty concentrating, as well as her reported difficulties in interpersonal relationships, could suggest generalized anxiety disorder. This diagnosis is also supported by her environment of ongoing familial instability and her personal negative self-perception.
3. **Bipolar Disorder**: Considering the familial history of bipolar disorder with the father, there is a genetic predisposition that might contribute to the development of bipolar disorder in the patient. The mix of depressive symptoms with potential episodes of increased appetite and overeating (possibly indicative of atypical depressive episodes or hypomanic episodes) warrants consideration of this diagnosis.

These diagnoses are complex and often require a comprehensive evaluation by a mental health professional, including a detailed patient history, physical examination, and possibly standardized mental health evaluations. It would be important for her to be evaluated by a psychiatrist or psychologist who can provide a thorough assessment and appropriate treatment plan.

Gemini Pro

Based on the information provided, here are the three most likely medical diagnoses that could be causing the patient's health problems:

1. Major Depressive Disorder (MDD):

- The patient's symptoms of frequent sadness, crying, increased appetite, guilt, low self-concept, anxiety, irritability, insomnia, hopelessness, and difficulty concentrating are highly suggestive of MDD.
- This diagnosis is further supported by the patient's family history of depression and anxiety.
- Additionally, the patient's difficulties in her interpersonal relationships, negative thoughts about her appearance and scholastic abilities, and guilt regarding her parents' marital problems are all common symptoms of MDD.

2. Generalized Anxiety Disorder (GAD):

- The patient's anxiety, difficulty concentrating, and insomnia are also consistent with GAD.
- Additionally, the patient's persistent negative thoughts about her appearance and scholastic abilities are often seen in individuals with GAD.
- The patient's family history of anxiety further supports this diagnosis.

3. Adjustment Disorder with Mixed Anxiety and Depressed Mood:

- This diagnosis is also a possibility, given the patient's recent stressors, including her parents' marital problems, academic difficulties, and social challenges.
- The patient's symptoms of sadness, anxiety, irritability, and difficulty concentrating are all consistent with this diagnosis.

Additional Considerations

- It is important to note that these are just the three most likely diagnoses and that other medical or psychological conditions could also be contributing to the patient's symptoms.
- A comprehensive evaluation by a qualified mental health professional is necessary to make a definitive diagnosis and develop an appropriate treatment plan.
- Additionally, the patient's medical history, including her asthma and overweight status, should be considered when developing a treatment plan.

Recommendations

- I recommend that the patient be referred to a mental health professional for a comprehensive evaluation and diagnosis.
- The mental health professional can then develop an appropriate treatment plan, which may include individual therapy, family therapy, medication, or a combination of these interventions.
- It is also important to address the patient's academic and social challenges. This may involve working with the school to provide her with additional support and resources, as well as helping her develop coping skills for dealing with stress and anxiety.

Important Note

- As a large language model, I cannot provide medical advice or diagnoses. The information provided above is for general knowledge and informational purposes only, and does not constitute medical advice. It is essential to consult with a qualified healthcare professional for any health concerns or before making any decisions related to your health or treatment.

Llama 3

Based on the symptoms presented, the three most likely medical diseases or diagnoses that could be contributing to the patient's health problem are:

1. **Major Depressive Disorder (MDD)**: The patient's symptoms of frequent sadness and crying, increased appetite and overeating, guilt, low self-concept, anxiety, irritability, insomnia, hopelessness, and difficulty concentrating are all consistent with a diagnosis of MDD. The patient's family history of depression and anxiety in her mother and bipolar disorder in her father also increase her risk for developing a mood disorder.

2. **Anxiety Disorder**: The patient's symptoms of anxiety, irritability, and insomnia suggest an underlying anxiety disorder, such as generalized anxiety disorder or social anxiety disorder. Her difficulties in interpersonal relationships and negative thoughts about her appearance and scholastic abilities may also be related to anxiety.

3. **Obstructive Sleep Apnea (OSA)**: The patient's symptoms of insomnia and daytime fatigue (implied by her difficulty concentrating) could be related to OSA, which is more common in overweight individuals like the patient. OSA can also contribute to depression, anxiety, and cognitive impairment.

It's essential to note that these diagnoses are not mutually exclusive, and the patient may be experiencing comorbid conditions. A thorough psychiatric evaluation and possible sleep study would be necessary to determine the underlying causes of the patient's symptoms.

Responses of chatbots to control vignette 5. depression

ChatGPT-4

Based on Brenda's history and symptoms, here's a possible ranking for the differential diagnosis:

1. **Unipolar Major Depressive Disorder**: Brenda's current symptoms are consistent with her previous episodes of major depression, which include mood changes, anxiety, sleep disturbance, and decreased appetite. Her history of remission with treatment and current exacerbation of symptoms fit well within this diagnosis.
2. **Generalized Anxiety Disorder (GAD)**: The symptoms of anxiety, pacing, and fidgeting, along with trouble sleeping, could suggest GAD. However, these could also be manifestations of her depressive disorder.
3. **Depression as a part of Bipolar Disorder**: Although Brenda has experienced multiple episodes of depression, there's no mention of any manic or hypomanic episodes. However, bipolar disorder should still be considered in the differential, as depressive episodes can precede the onset of manic symptoms.
4. **Substance-induced Mood Disorder**: This is less likely given the information, but should be considered if there's any undisclosed substance use that could be contributing to mood changes. Brenda's reference to a friend's overdose could hint at awareness of substance risks but does not directly imply her own use.
5. **Attention-Deficit Hyperactivity Disorder (ADHD)**: While sleep disturbances and fidgeting are common in ADHD, Brenda's primary issues seem to center around mood disturbances more characteristic of depressive disorders. ADHD is thus less likely to be the primary concern but could be a comorbid condition.

It's crucial to continue evaluating Brenda's history and symptoms, and potentially to conduct further diagnostic assessments to refine this differential diagnosis.

Gemini Pro

Based on the information provided, here's my assessment of Brenda's potential diagnoses, ranked in order of likelihood:

1. Unipolar Major Depressive Disorder (MDD):

- **Strong Evidence:**
  - Two previous episodes of MDD with clear remissions.
  - Current symptoms align with MDD criteria: depressed mood, anhedonia, weight loss, sleep disturbance, fatigue, feelings of worthlessness.
  - No evidence of manic or hypomanic episodes suggestive of bipolar disorder.

2. Depression as part of Bipolar Disorder:

- **Possible Evidence:**
  - History of two previous depressive episodes, potentially indicating a recurrent pattern.
  - Current symptoms of anxiety and agitation could be indicative of bipolarity.
  - However, no clear evidence of manic or hypomanic episodes is present.

3. Substance-Induced Mood Disorder:

- **Unlikely:** No mention of substance use or abuse in the provided information.

4. Generalized Anxiety Disorder (GAD):

- **Possible:** Brenda reports experiencing anxiety and restlessness. However, the prominent presence of depressed mood and other MDD symptoms makes GAD less likely as the primary diagnosis.

5. Attention-Deficit Hyperactivity Disorder (ADHD):

- **Unlikely:** No information suggests symptoms of inattention, hyperactivity, or impulsivity.

Overall:
Based on the available information, Unipolar Major Depressive Disorder appears to be the most likely diagnosis for Brenda. However, further evaluation is crucial to rule out Bipolar Disorder definitively and assess the potential presence of co-occurring anxiety.

Recommendations:

- Conduct a thorough assessment to explore the presence and severity of manic or hypomanic symptoms to clarify the Bipolar Disorder possibility.
- Evaluate the potential impact of anxiety on Brenda's overall presentation.
- Consider additional information like family history, current stressors, and past treatment responses to refine the diagnosis and develop an appropriate treatment plan.

Llama 3

Based on the information provided, here is my ranking of the differential diagnosis in order of likelihood:

1. **Unipolar major depressive disorder**: This is the most likely diagnosis given Brenda's history of two previous episodes of major depression, which remitted with treatment. Her current symptoms, such as trouble sleeping, anxiety, crying, decreased appetite, weight loss, and feelings of worthlessness, are all consistent with a major depressive episode.

2. **Depression as a part of bipolar disorder**: Although Brenda has had two previous episodes of depression, there is no mention of manic or hypomanic episodes in her history. However, it is possible that she may have undiagnosed bipolar disorder, which could be contributing to her current symptoms.

3. **Generalized anxiety disorder**: Brenda's symptoms of anxiety, pacing, and fidgeting suggest that she may also be experiencing an anxiety disorder. However, her primary complaint is depression, and her anxiety symptoms seem to be secondary to her depressive episode.

4. **Attention-deficit hyperactivity disorder (ADHD)**: While Brenda mentions pacing and fidgeting, which could be symptoms of ADHD, these symptoms are more likely related to her anxiety and depression in this context. There is no other evidence to suggest ADHD.

5. **Substance-induced mood disorder**: There is no indication in the patient's history that substance use is contributing to her depressive symptoms. In fact, she explicitly denies suicidal thoughts and plans, which suggests that she is not using substances as a means of coping with her depression.

Please note that a comprehensive diagnostic assessment would require further evaluation and consideration of other potential diagnoses.

Responses of chatbots to control vignette 6. Social Anxiety Disorder

ChatGPT-4

The description provided suggests several possible medical diagnoses that could be considered for the 26-year-old student experiencing these symptoms:

1. **Social Anxiety Disorder (SAD)**: This is likely the primary diagnosis to consider given the specific symptoms of intense anxiety, palpitations, trembling, blushing, and sweating in social interactions or performance situations, and significant distress or impairment in social, occupational, or other important areas of functioning.
2. **Generalized Anxiety Disorder (GAD)**: While the symptoms are primarily triggered by social situations, it's possible that the student experiences pervasive anxiety in other aspects of life too, which could be indicative of GAD.
3. **Panic Disorder**: This might be considered if the symptoms like palpitations and trembling occur in intense episodes that peak within minutes (panic attacks). If these episodes are followed by persistent concern about having another attack or the implications of the attack, this diagnosis might be applicable.

It's important that the individual seeks a comprehensive evaluation from a mental health professional to accurately diagnose and treat the condition based on a detailed history and assessment.

Gemini Pro

Based on the information provided, the three most likely medical diagnoses for the 26-year-old student are:

1. Social Anxiety Disorder (SAD): This is the most likely diagnosis given the patient's symptoms and history. SAD is characterized by a persistent fear of social situations where the individual is exposed to possible scrutiny by others. This fear can lead to significant anxiety, avoidance of social situations, and distress. The patient's description of feeling anxious in presentations, exams, and meetings with authority figures aligns with common triggers for SAD. Additionally, his avoidance of social contact and feelings of loneliness are also consistent with this diagnosis.
2. Specific Phobia: While less likely than SAD, specific phobia is another possibility. Specific phobias involve an intense and irrational fear of a specific object or situation, in this case, social situations involving performance or interaction with authority figures. The patient's symptoms of anxiety, physical symptoms, and avoidance behavior are characteristic of specific phobia. However, the broader range of social fears suggests SAD as the more likely diagnosis.
3. Generalized Anxiety Disorder (GAD): Although less likely than the previous two, GAD could also be considered. GAD is characterized by excessive and uncontrollable worry about various aspects of life. The patient's anxiety about presentations, exams, and authority figures could be

part of a broader pattern of excessive worry. However, the specific social focus and avoidance behavior point more towards SAD or specific phobia.

Additional Considerations and Recommendations:

- A thorough clinical evaluation by a mental health professional is crucial to confirm the diagnosis and rule out other possible causes. This evaluation should include a detailed history, mental status examination, and potentially psychological testing.
- Comorbid conditions like depression should be considered and addressed if present.
- Treatment options for the identified diagnoses may include:
  - Psychotherapy, such as Cognitive Behavioral Therapy (CBT), which helps individuals identify and change unhelpful thought patterns and behaviors.
  - Medication, such as selective serotonin reuptake inhibitors (SSRIs), which can help manage anxiety symptoms.
  - Social skills training and exposure therapy, which can help individuals gradually confront their fears and develop coping mechanisms.

It is important to note that this information is for educational purposes only and should not be used for self-diagnosis or treatment. Only a qualified healthcare professional can make an accurate diagnosis and recommend appropriate treatment.

Llama 3

Based on the symptoms described in the vignette, the three most likely medical diseases or diagnoses that could be causing this health problem are:

1. **Social Anxiety Disorder (SAD)**: Also known as social phobia, this is a mental health disorder characterized by excessive and persistent fear of social or performance situations in which the person is exposed to possible scrutiny by others. The symptoms described in the vignette, such as anxiety in specific situations, physical symptoms like palpitations and sweating, and avoidance of social contact, are all consistent with SAD.

2. **Panic Disorder**: This is an anxiety disorder characterized by recurring panic attacks, which are sudden feelings of intense fear or discomfort that reach a peak within minutes. The physical symptoms described in the vignette, such as palpitations, tremors, and sweating, are similar to those experienced during a panic attack. However, the patient's symptoms seem to be more situation-specific and related to social anxiety, which makes SAD a more likely diagnosis.

3. **Generalized Anxiety Disorder (GAD)**: This is a mental health disorder characterized by excessive and persistent worry about everyday things, even when there is no apparent reason to worry. While GAD can manifest with physical symptoms like palpitations and sweating, it typically involves more general and free-floating anxiety rather than situation-specific fears like those described in the vignette. However, it is possible that the patient's social anxiety is part of a broader pattern of generalized anxiety.

It's worth noting that these diagnoses are not mutually exclusive, and it's possible for the patient to have features of multiple anxiety disorders. A thorough diagnostic evaluation by a qualified

healthcare professional would be necessary to determine the most accurate diagnosis and develop an appropriate treatment plan.

Responses of chatbots to control vignette 7. Panic disorder

ChatGPT-4

Based on the description provided, Dave's symptoms and experiences suggest a few potential diagnoses:

1. **Panic Disorder**: Dave's episodes of intense fear that peak rapidly along with physical symptoms such as sweating, heart palpitations, chest pain, and difficulty breathing are characteristic of panic attacks. His persistent worry about having another attack and behavioral changes like avoiding places and activities due to fear of these attacks further support a diagnosis of panic disorder.
2. **Agoraphobia**: Alongside panic disorder, Dave may be experiencing agoraphobia. This condition involves a fear of being in situations where escape might be difficult or help wouldn't be available if things go wrong. Dave's avoidance of unfamiliar places and social situations suggests this could be a contributing factor.
3. **Generalized Anxiety Disorder (GAD)**: Although the primary symptoms align more with panic disorder and agoraphobia, Dave's persistent worry and stress about future panic attacks and other aspects of life could also indicate generalized anxiety disorder, which is characterized by chronic anxiety, exaggerated worry, and tension, often without an obvious cause.

It's important for Dave to continue working with his healthcare provider to thoroughly explore these possibilities and receive appropriate treatment, which may include therapy, medication, or a combination of both.

Gemini Pro

Based on the information provided, here are the three most likely medical diagnoses that could be causing Dave's symptoms:

1. Panic Disorder: This is the most likely diagnosis given Dave's recurrent and unexpected panic attacks, accompanied by intense fear, physical symptoms like sweating, heart palpitations, chest pain, and shortness of breath, and the fear of dying. Additionally, his avoidance of situations where escape might be difficult or help unavailable aligns with the typical pattern of panic disorder.
2. Generalized Anxiety Disorder (GAD): While Dave's primary concern seems to be the panic attacks, he also describes persistent worry about having another attack, which could indicate GAD. GAD is characterized by excessive anxiety and worry about various aspects of life, often accompanied by physical symptoms like fatigue, restlessness, and difficulty concentrating.
3. Social Anxiety Disorder (SAD): Although less likely than the first two diagnoses, Dave's avoidance of social situations and unfamiliar places could also suggest SAD. This disorder involves a significant fear of social situations due to the worry of being judged or negatively

evaluated by others. The fear can lead to avoidance of social events and interactions, which aligns with Dave's current behavior.

Important Note:

It's crucial to remember that this information is not a definitive diagnosis and should not be used as a substitute for professional medical advice. Only a qualified healthcare professional can accurately diagnose Dave's condition after a thorough evaluation, including a physical examination, psychological assessment, and potentially additional tests.

It's important for Dave to seek professional help to address his symptoms and improve his quality of life. With proper treatment, which may include therapy, medication, or a combination of both, Dave can manage his anxiety and panic attacks effectively and regain control over his life.

Llama 3

Based on the symptoms described, the three most likely medical diseases or diagnoses that could be causing Dave's health problem are:

1. **Panic Disorder**: This is a mental health condition characterized by recurring panic attacks, which are sudden feelings of intense fear or anxiety that reach a peak within minutes. The symptoms described, such as sweating, heart palpitations, chest pain, and shortness of breath, are consistent with panic attacks. Additionally, the persistent worry about having another attack and avoidance behaviors (e.g., avoiding unfamiliar places) are also characteristic of panic disorder.

2. **Anxiety Disorder (Generalized Anxiety Disorder or Social Anxiety Disorder)**: While panic disorder is a strong possibility, it's also possible that Dave is experiencing generalized anxiety or social anxiety disorder. These conditions can cause excessive and persistent worry, fear, or anxiety about everyday things, social situations, or specific events. The avoidance behaviors and impact on daily life (e.g., turning down social invitations) could be indicative of an anxiety disorder.

3. **Cardiac Arrhythmia or other Cardiac Condition**: Although Dave's primary care physician was unable to find a medical explanation for his symptoms, it's possible that an underlying cardiac condition, such as a cardiac arrhythmia (abnormal heartbeat), could be contributing to his symptoms. The chest pain and discomfort, heart palpitations, and shortness of breath could be indicative of a cardiac issue. A thorough cardiac evaluation would be necessary to rule out any potential cardiac conditions.

It's essential to note that a comprehensive diagnostic evaluation by a qualified healthcare professional is necessary to determine the underlying cause of Dave's symptoms. A mental health professional, such as a psychologist or psychiatrist, may also be involved in the diagnostic process to assess for potential mental health conditions.
